# RhoBTB1 reverses established arterial stiffness in angiotensin II–induced hypertension by promoting actin depolymerization

**DOI:** 10.1172/jci.insight.158043

**Published:** 2022-05-09

**Authors:** Shi Fang, Jing Wu, John J. Reho, Ko-Ting Lu, Daniel T. Brozoski, Gaurav Kumar, Alec M. Werthman, Sebastiao Donato Silva, Patricia C. Muskus Veitia, Kelsey K. Wackman, Angela J. Mathison, Bi Qing Teng, Chien-Wei Lin, Frederick W. Quelle, Curt D. Sigmund

**Affiliations:** 1 Department of Physiology and Cardiovascular Center, Medical College of Wisconsin, Milwaukee, Wisconsin, USA.; 2 Department of Neuroscience and Pharmacology, Carver College of Medicine, University of Iowa, Iowa City, Iowa, USA.; 3 Genomic Sciences and Precision Medicine Center;; 4 Division of Research, Department of Surgery; and; 5 Division of Biostatistics, Medical College of Wisconsin, Milwaukee, Wisconsin, USA.

**Keywords:** Vascular Biology, Hypertension

## Abstract

Arterial stiffness predicts cardiovascular disease and all-cause mortality, but its treatment remains challenging. Mice treated with angiotensin II (Ang II) develop hypertension, arterial stiffness, vascular dysfunction, and a downregulation of Rho-related BTB domain–containing protein 1 (RhoBTB1) in the vasculature. RhoBTB1 is associated with blood pressure regulation, but its function is poorly understood. We tested the hypothesis that restoring RhoBTB1 can attenuate arterial stiffness, hypertension, and vascular dysfunction in Ang II–treated mice. Genetic complementation of RhoBTB1 in the vasculature was achieved using mice expressing a tamoxifen-inducible, smooth muscle–specific RhoBTB1 transgene. RhoBTB1 restoration efficiently and rapidly alleviated arterial stiffness but not hypertension or vascular dysfunction. Mechanistic studies revealed that RhoBTB1 had no substantial effect on several classical arterial stiffness contributors, such as collagen deposition, elastin content, and vascular smooth muscle remodeling. Instead, Ang II increased actin polymerization in the aorta, which was reversed by RhoBTB1. Changes in the levels of 2 regulators of actin polymerization, cofilin and vasodilator-stimulated phosphoprotein, in response to RhoBTB1 were consistent with an actin depolymerization mechanism. Our study reveals an important function of RhoBTB1, demonstrates its vital role in antagonizing established arterial stiffness, and further supports a functional and mechanistic separation among hypertension, vascular dysfunction, and arterial stiffness.

## Graphical abstract

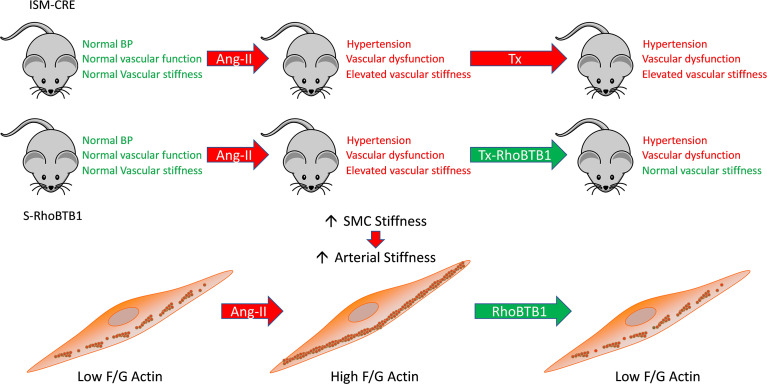

## Introduction

Cardiovascular disease is the leading cause of death in the United States (1). The American Heart Association estimates that cardiovascular disease will cost more than $1.1 trillion by 2035 (2), let alone the added cost burden associated with the COVID-19 pandemic (3). Hypertension is one of the most pronounced preventable risk factors for cardiovascular disease and is commonly associated with many forms of end-organ damage, including endothelial dysfunction and increased arterial stiffness.

Arterial stiffness predicts both cardiovascular and all-cause mortality (4). It is well established that mechanical factors such as increased wall stress, caused by high BP; biochemical factors including oxidative stress; and inflammation associated with aging, diabetes, and activation of the renin-angiotensin system contribute to the development of arterial stiffness (5, 6). In response to these challenges, blood vessels undergo a collection of structural modifications, for instance, increased deposition and cross-linking of extracellular matrix (ECM) (7, 8). Vascular smooth muscle cells (SMCs) also promote arterial stiffness by proliferation, calcification, and increase of cellular stiffness (9, 10).

The nuclear receptor transcriptional factor PPARγ is an important regulator of vascular function. As reviewed recently, PPARγ in endothelium increases the bioavailability of NO, whereas its activity in vascular smooth muscle is required for the responsiveness to NO (11). One of the transcriptional targets of PPARγ is Rho-related BTB domain–containing protein 1 (*Rhobtb1*), an atypical Rho GTPase whose molecular and physiological importance remains only partially understood. *RHOBTB1* was identified in a GWAS to be associated with diastolic BP and to be what is termed an interacting locus in a study of more than 1 million people (12, 13). RhoBTB1 deficiency was identified in mice expressing an SMC-specific, dominant-negative, PPARγ mutation identified in humans, which inhibits normal PPARγ activity (14). These mice exhibited severely impaired vascular function, hypertension, and increased arterial stiffness (14, 15). We recently showed that restoring *Rhobtb1* expression in vascular SMCs in mice with SMC-specific, dominant-negative PPARγ reversed the hypertension, vascular dysfunction, and arterial stiffness caused by the smooth muscle–specific expression of a dominant-negative mutation of PPARγ (14, 16). However, whether RhoBTB1 reversed the arterial stiffness directly by altering the material stiffness or indirectly by lowering BP and improving vasodilation is unknown. Moreover, the generality of the protective effects of RhoBTB1 remains to be understood.

In this study, we assessed whether the cardioprotective effect of RhoBTB1 is active in other forms of hypertension and arterial stiffness. To do this, we capitalized on the observation that *Rhobtb1* expression is decreased by angiotensin II (Ang II). Genetic complementation was used to restore *Rhobtb1* expression in mice infused with Ang II. This study was designed as a reversal study, that is, we tested the effectiveness of RhoBTB1 to reverse established hypertension, just as pharmacological agents are used to treat a patient presenting with hypertension. Interestingly, restoring RhoBTB1 specifically in vascular SMCs effectively and rapidly reversed Ang II–established arterial stiffness but did not lower BP or improve vascular function. Further evaluation revealed that RhoBTB1 had little effect on classical mechanisms implicated in arterial stiffness, such as collagen and elastin deposition, and did not affect the overall structure of the vessel. On the contrary, RhoBTB1 restoration decreased actin-filament organization in the vasculature. These observations suggest that RhoBTB1 may be a new therapeutic target for arterial stiffness. Conceptually, our data also indicate that arterial stiffness can be regulated independently of BP.

## Results

**Figure 1:**
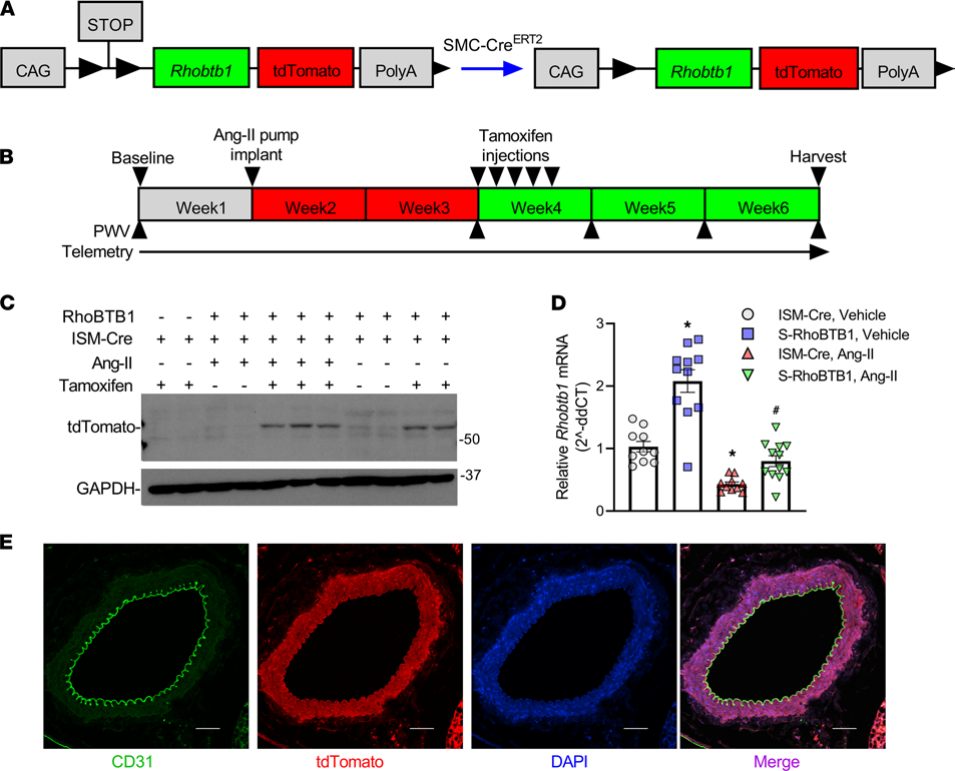
Mouse model and protocol. (**A**) S-RhoBTB1 mice expressing a Tx-inducible, smooth muscle–specific *Rhobtb1* transgene were obtained by breeding ISM-Cre mice, which express Tx-inducible, smooth muscle–specific Cre-recombinase, and RhoBTB1^IND^ mice, which express a LoxP-STOP-LoxP-*Rhobtb1*-tdTomato transgene. Cre-recombinase catalyzes the removal of the LoxP-STOP-LoxP cassette, inducing expression of *Rhobtb1* and tdTomato. (**B**) ISM-Cre mice and S-RhoBTB1 mice received either vehicle or Ang II treatment via osmotic minipumps for 5 weeks. All animals received 5 consecutive daily Tx injections 2 weeks after pump implantation. BP was continuously measured by radiotelemetry from 1 week before pump implantation until the end of the protocol. PWV was measured before pump implantation, 2 weeks after pump implantation, and at 0, 1, and 2 weeks after the completion of Tx injections. Tissues were harvested at the end of the protocol. (**C**) Representative Western blot detecting tdTomato and GAPDH in aorta (*n* = 2–3 biological replicates). Mice that did not receive Tx received the corn oil vehicle. Positions of actual size markers are shown. (**D**) Relative *Rhobtb1* mRNA levels in aorta from the indicated animals (*n* = 10–12, as indicated in the dot plots). *18S* rRNA was used as an internal control. ISM-Cre mice treated with vehicle were used as controls to calculate 2^ΔΔCT^ for *Rhobtb1*. (**E**) Immunostaining of aorta from S-RhoBTB1 mice. Green indicates the endothelium marker CD31; red indicates reporter gene tdTomato; blue indicates the nuclei staining with DAPI. Scale bar: 100 μm (*n* = 3 biological replicates). All data are presented as a mean ± SEM. Two-way ANOVA with Tukey’s multiple comparisons were used for statistical analysis. *P < 0.05 versus ISM-Cre, vehicle. ^#^*P* < 0.05 vs ISM-Cre Ang II.

For this study, we used S-RhoBTB1 mice that express a tamoxifen-inducible (Tx-inducible), smooth muscle–specific RhoBTB1 transgene (Figure 1A). Cre-mediated recombination excises the LoxP-STOP-LoxP signal and induces the expression of both *Rhobtb1* and tdTomato. ISM-Cre mice that express Tx-inducible, smooth muscle–specific Cre-recombinase without *Rhobtb1* transgene served as controls. All mice expressed Cre-recombinase and were treated with Tx.

We developed a reversal protocol in which ISM-Cre and S-RhoBTB1 littermates received either vehicle or Ang II infusion via osmotic minipump (490 ng/kg/min for 6 weeks), followed by 5 consecutive daily Tx injections 2 weeks after Ang II–induced hypertension was established (Figure 1B). Tissue was harvested 3 weeks after the initiation of Tx injections. Western blotting results for the reporter gene, tdTomato, in aorta lysates suggest that the transgene in S-RhoBTB1 mice was successfully activated by Tx (Figure 1C). Ang II had no appreciable effect on transgene activation. Expression of tdTomato was restricted to vascular SMCs in aorta and was not detected in endothelium (Figure 1E). There was no appreciable expression of tdTomato in heart and kidney (data not shown). In the absence of Ang II, expression of *Rhobtb1* mRNA was significantly increased in S-RhoBTB1 mice, compared with ISM-Cre mice (Figure 1D). The *Rhobtb1* mRNA level was significantly decreased after Ang II treatment of ISM-Cre mice but, notably, was restored to baseline levels in Ang II–treated S-RhoBTB1 mice. Thus, the level of *Rhobtb1* expression in Ang II–treated S-RhoBTB1 mice was normal, not overexpressed.

**Figure 2:**
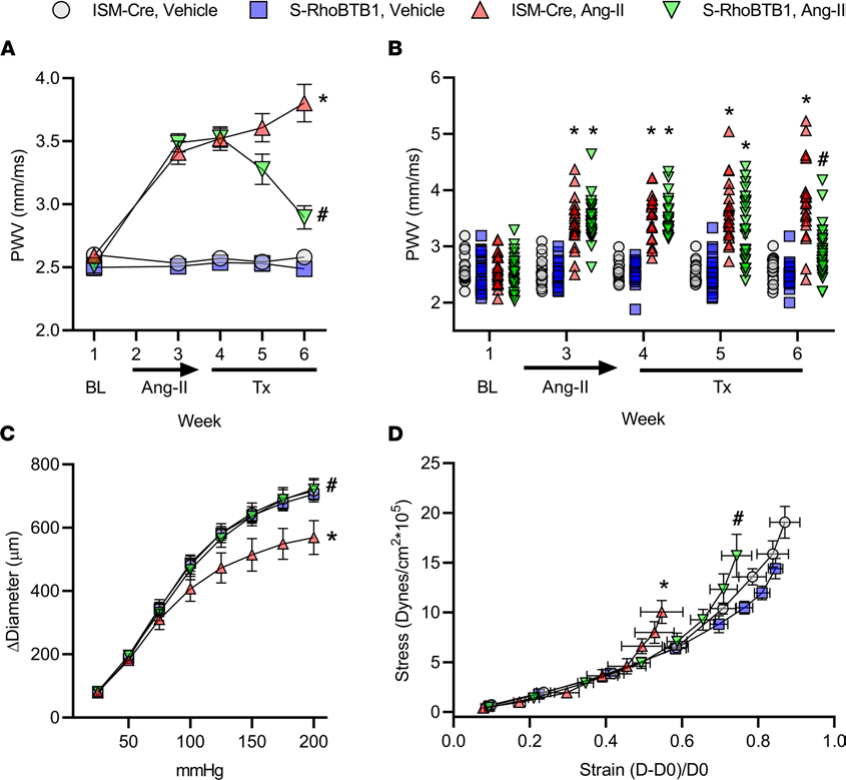
Arterial stiffness. (**A** and **B**) PWV was measured at baseline (BL) before Ang II treatment, 2 weeks after Ang II pump implantation (week 3), and 0, 1, and 2 weeks (weeks 4–6) after completion of Tx injections (**A**); *n* = 20–27, as indicated in dot plots in **B**. (**B**) Individual PWV recordings at each time point from **A** are shown. (**C** and **D**) Aortic compliance was evaluated by the pressure-diameter (**C**) and stress-strain relationships (**D**). Sample numbers are as follows: ISM-CRE vehicle, *n* = 6; S-RhoBTB1 vehicle, *n* = 9; ISM-Cre Ang II, *n* = 6; S-RhoBTB1 Ang II, *n* = 9.All data are presented as a mean ± SEM. Two-way ANOVA with Tukey’s multiple comparisons were used for PWV analysis. Two-way ANOVA with repeated measurements were used for pressure-diameter and stress-strain relationships. *P < 0.05 vs. ISM-Cre, vehicle; ^#^*P* < 0.05 vs. ISM-Cre, Ang II.

To test whether RhoBTB1 restoration is sufficient to ameliorate the increased arterial stiffness caused by Ang II, we first measured aortic pulse wave velocity (PWV) at the time points shown in Figure 1B: baseline (week 1, before Ang II pump implantation), 2 weeks after Ang II infusion (week 3), 2 days after Tx administration (week 4), and 1 (week 5) and 2 (week 6) weeks after Tx administration. RhoBTB1 had no effect on arterial stiffness in ISM-Cre or S-RhoBTB1 mice that were vehicle treated (Figure 2A). Ang II infusion markedly increased PWV in both ISM-Cre and S-RhoBTB1 mice before Tx administration, signifying the establishment of arterial stiffness. Remarkably, PWV started to decline 1 week after Tx administration and nearly returned to baseline 2 weeks after Tx treatment in Ang II–treated S-RhoBTB1 mice.

Evaluation of the individual animal data demonstrated the robustness of the reversal in arterial stiffness in response to RhoBTB1 (Figure 2B). The large sample size is an indicator of the rigor of our study, in which we measured PWV in all cohorts of mice that underwent a terminal mechanistic evaluation to ensure phenotypic consistency. Similarly, both pressure-diameter (Figure 2C) and stress-strain (Figure 2D) curves, performed in the absence of calcium, suggest that aortic compliance was decreased by Ang II and was normalized upon RhoBTB1 restoration. Again, modest overexpression of *Rhobtb1* did not affect aortic compliance in vehicle-treated mice.

**Figure 3:**
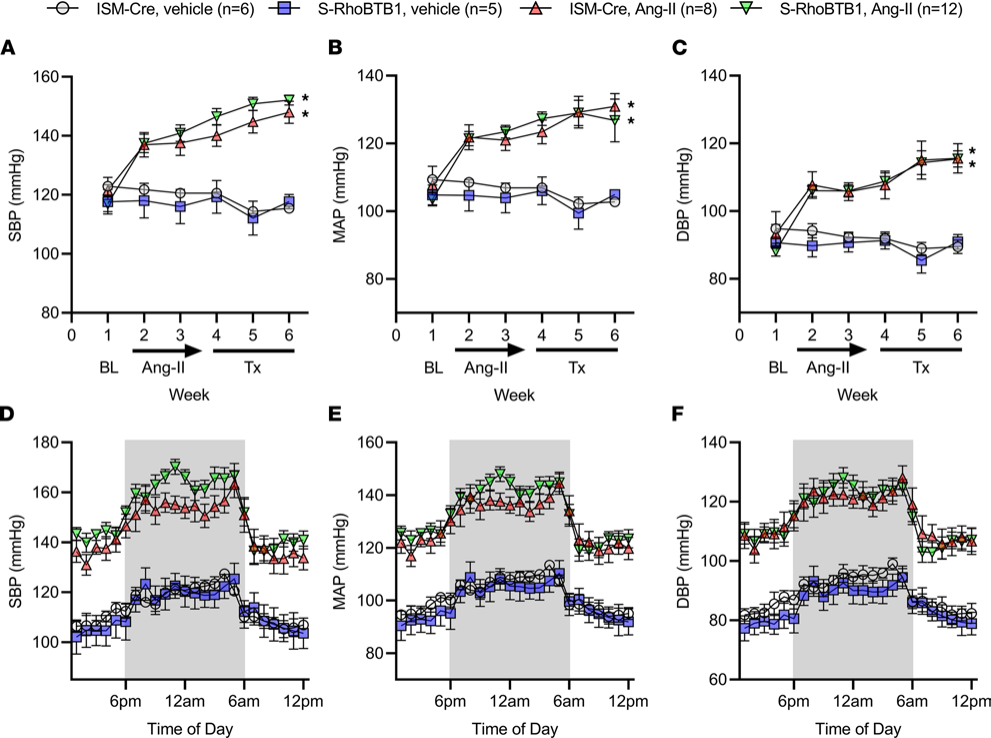
Blood pressure. BP was continuously measured by radiotelemetry (sample numbers are as follows: ISM-CRE vehicle, *n* = 6; S-RhoBTB1 vehicle, *n* = 5; ISM-Cre Ang II, *n* = 8; S-RhoBTB1 Ang II, *n* = 12.). (**A**–**C**) Systolic BP (SBP, **A**), mean arterial BP (MAP, **B**), and diastolic BP (DBP, **C**) during the entire protocol are presented as weekly averages. (**D**–**E**)Hourly SBP (**D**), MAP (**E**), and DBP (**F**) were analyzed during the last week of the protocol. All data are presented as mean ± SEM. Two-way ANOVA with Tukey’s multiple comparisons were used for data analysis at each time point. *P < 0.05 vs. ISM-Cre, vehicle.

Next, we sought to determine whether the reduction in arterial stiffness is secondary to lowered BP. Continuous radiotelemetry recordings of BP showed that Ang II increased the systolic, mean, and diastolic BPs in ISM-Cre and in S-RhoBTB1 mice to a similar extent before Tx administration (Figure 3, A–C). Unlike the reversal of arterial stiffness, restoring RhoBTB1 did not lower systolic, mean, or diastolic BPs. Analysis of hourly BP during the final week of the experiment revealed a preservation of the normal circadian rhythm of BP (Figure 3, D–F). Moreover, there were no pronounced effects of RhoBTB1 activation on pulse pressure, heart rate, and activity (Supplemental Figure 1; supplemental material available online with this article; https://doi.org/10.1172/jci.insight.158043DS1). Despite lowered arterial stiffness, pulse pressure trended to be higher in Ang II–treated S-RhoBTB1 mice than in ISM-Cre mice during the last week of the study (Supplemental Figure 1D). Thus, we concluded that the improvement in arterial stiffness was not mediated by a reduction in BP and occurred despite the preservation of hypertension.

**Figure 4:**
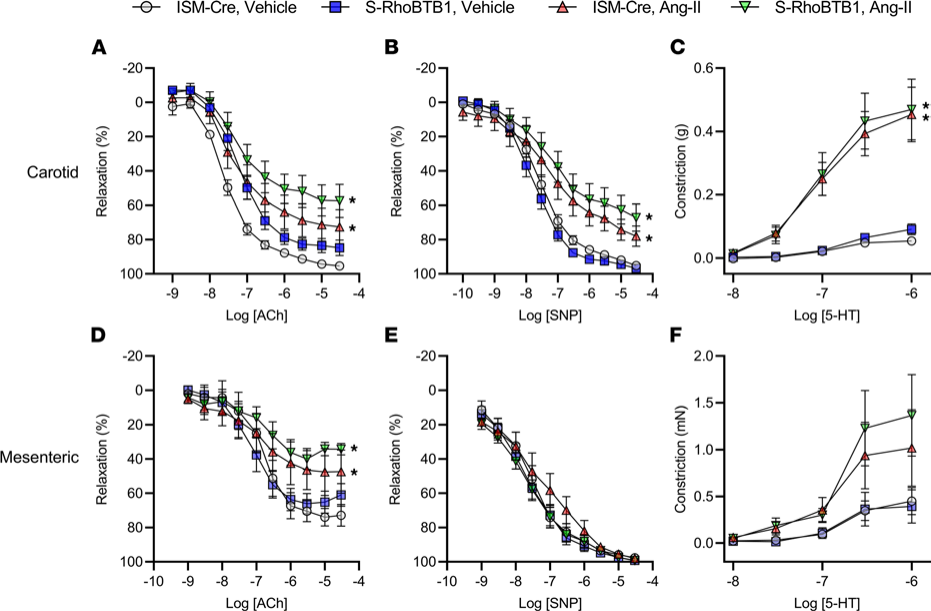
Vascular reactivity. (**A**–**C**) Vascular reactivity was studied using wire myography on carotid arteries (sample numbers are as follows: ISM-CRE vehicle, *n* = 6; S-RhoBTB1 vehicle, *n* = 6; ISM-Cre Ang II, *n* = 9; S-RhoBTB1 Ang II, *n* = 11); and (**D**–**F**) mesenteric arteries (sample numbers are as follows: ISM-CRE vehicle, *n* = 6; S-RhoBTB1 vehicle, *n* = 9; ISM-Cre Ang II, *n* = 5; S-RhoBTB1 Ang II, *n* = 9.) (**A** and **D**). ACh-mediated vasodilation was used to evaluate endothelial-dependent vascular relaxation. (**D**) One mesenteric artery sample was excluded because its reading fell out of the cutoff value predetermined for quality control. (**B** and **E**) Sodium nitroprusside–mediated (SNP-mediated) vasodilation was used to evaluate endothelium-independent vascular relaxation. (**C** and **F**) Serotonin-mediated (5-HT–mediated) vasoconstriction was measured. All data are presented as a mean ± SEM. *P < 0.05 vs. ISM-Cre, vehicle. Two-way ANOVA with repeated measurements were used for statical analysis. The E_max_ values for each biological replicate are provided in Supplemental Figure 2. E_max_, maximum effect.

Ex vivo analysis of carotid arteries from Ang II–treated ISM-Cre mice showed impaired vasodilation responses to acetylcholine (ACh) and sodium nitroprusside, as well as enhanced vasoconstriction to serotonin (Figure 4, A–C). There was no improvement in ACh- or sodium nitroprusside–mediated vasodilation in Ang II–treated S-RhoBTB1 mice when compared with Ang II–treated ISM-Cre mice. However, it is worth noting the maximum effect (E_max_) for both ACh and sodium nitroprusside in the carotid artery was significantly decreased compared with all other groups, suggesting a potential worsening of the phenotype (Supplemental Figure 2). Similarly, the impaired vasodilation responses observed in mesenteric resistance arteries were not improved by RhoBTB1 (Figure 4, D–F, and Supplemental Figure 2). Thus, restoring RhoBTB1 in vascular SMCs did not rescue the impaired vascular reactivity Ang II caused and perhaps worsened it.

The BP and vascular reactivity data suggested that the attenuation of Ang II–established arterial stiffness by RhoBTB1 restoration was not a mere consequence of either lowered BP or improved vasodilation. Therefore, we investigated whether RhoBTB1 decreased arterial stiffness by directly regulating the properties of the vascular wall.

**Figure 5:**
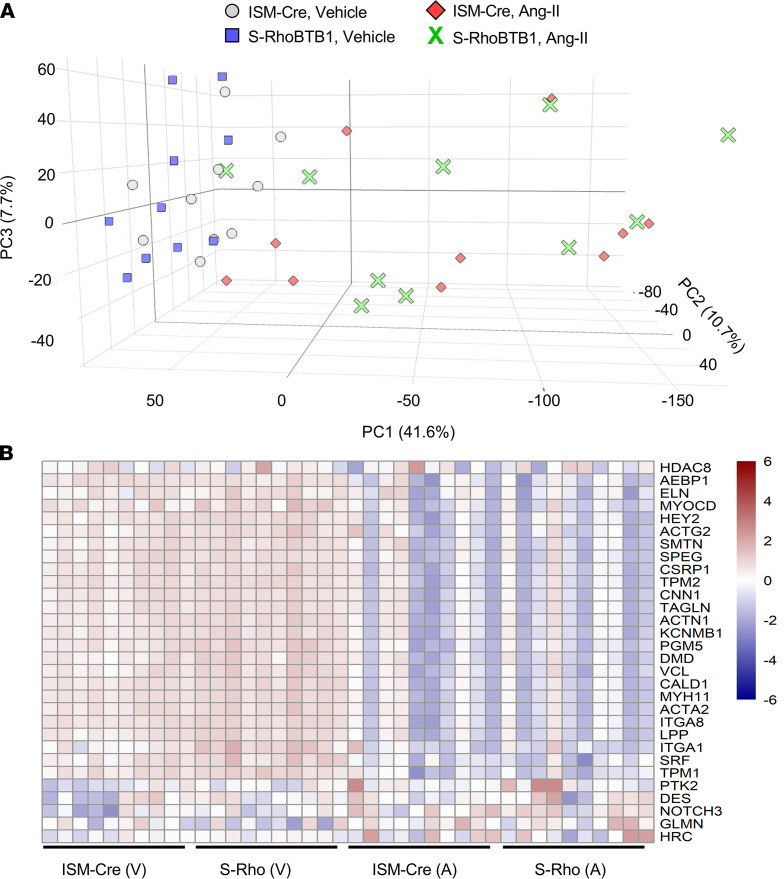
PCA and vascular SMC differentiation markers. (**A**) PCA of global gene expression in the RNA-Seq data set (*n* = 10). (**B**) Transactivation profiles of vascular smooth muscle differentiation markers in each sample.

To understand the mechanisms by which RhoBTB1 reversed arterial stiffness, we simultaneously examined the transcriptome of the aorta and focused on the major known contributors to arterial stiffness. Bulk RNA-Seq was performed using whole aorta from all 4 groups of mice (Gene Expression Omnibus accession series GSE189003; Supplemental Table 1). Principal component analysis (PCA) revealed that the vehicle-treated ISM-Cre and S-RhoBTB1 mice were closely clustered, suggesting sufficient quality in the reproducibility of the control samples (Figure 5A). On the contrary, samples from Ang II–treated ISM-Cre mice and S-RhoBTB1 mice were equally dispersed along the PC1 and PC3 axes, suggesting a highly variable transcriptome response to Ang II. The reason for this is unclear; perhaps the response was caused by 1 or more of the many mechanisms by which Ang II can cause hypertension. Moreover, an excellent correlation between the level of *Rhobtb1* expression detected by RNA-Seq and quantitative real-time PCR (RT-PCR) in all groups suggested that RNA-Seq can be used to predict the transactivation of the genes of interest (Supplemental Figure 3).

**Figure 6:**
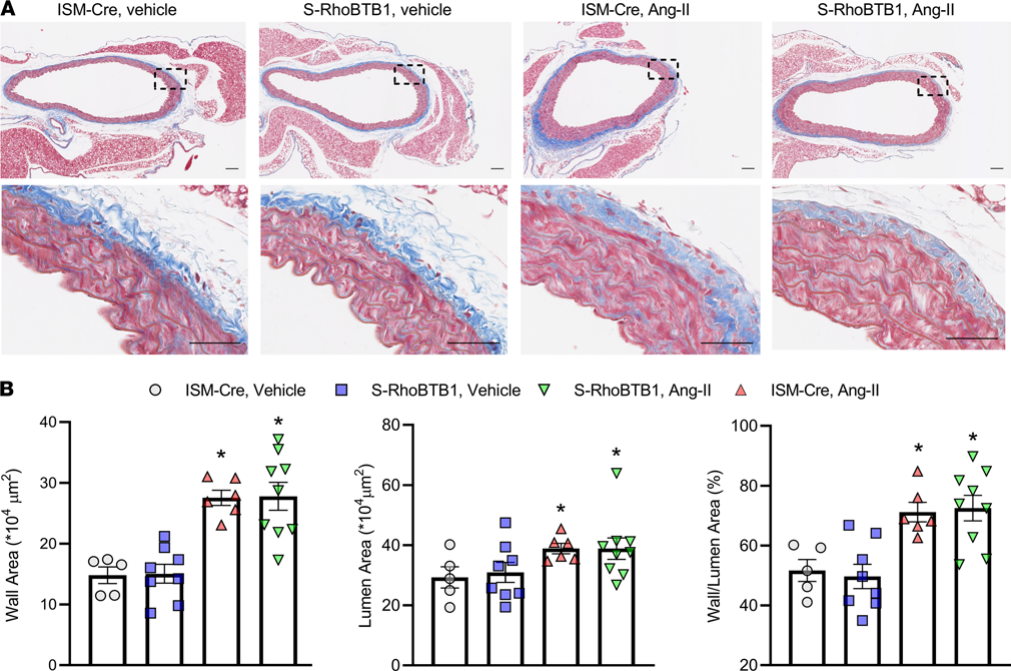
Vascular SMC remodeling. (**A**) Masson’s trichrome staining of aorta cross-sections from indicated animals 2 weeks after the completion of Tx injections (*n* = 5–9, as indicated in the dot plots in **B**. Scale bar: 100 μm. (**B**) Wall area, lumen area, and wall area to lumen area ratio from Masson’s trichrome staining were used to evaluate vascular smooth muscle remodeling. All data are presented as mean ± SEM. Two-way ANOVA with Tukey’s multiple comparisons were used for data analysis. *P < 0.05 vs. ISM-Cre, vehicle.

Ang II infusion changed the expression of classical markers for differentiated vascular SMCs, downregulating many and upregulating some (17) (Figure 5B). However, no differentially expressed genes were identified among these markers of vascular SMC differentiation between Ang II–treated S-RhoBTB1 and Ang II–treated ISM-Cre mice, suggesting RhoBTB1 did not reprogram the vascular SMC phenotype. Consistently, the expression level of *Klf4*, a transcription factor that regulates vascular SMC differentiation, was downregulated by Ang II but was unaffected by RhoBTB1 restoration (Supplemental Figure 4). We then measured vascular dimensions to determine if there was any evidence of vascular remodeling (Figure 6A). Although Ang II increased wall area, lumen area, and wall/lumen ratio, there was no improvement of these parameters after RhoBTB1 restoration (Figure 6B).

**Figure 7:**
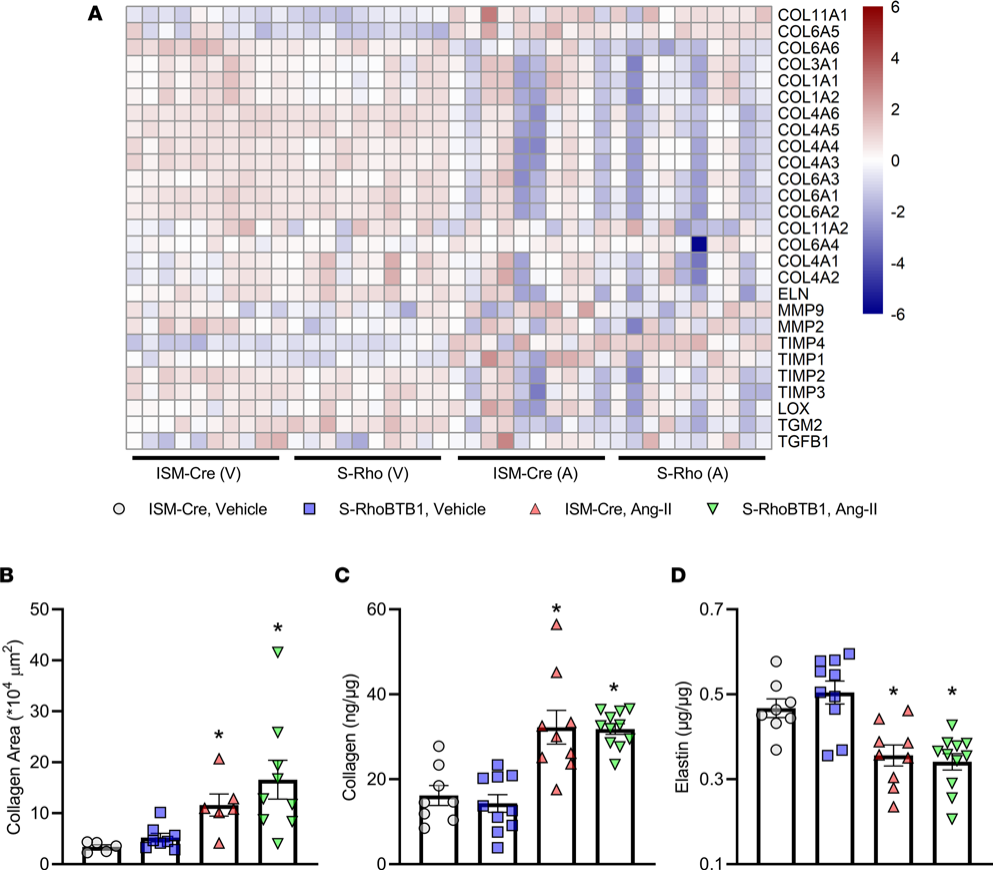
Extracellular matrix. (**A**) Transactivation profiles of classical ECM components contributing to arterial stiffness such as collagen, elastin, matrix metalloproteinase, tissue inhibitors of metalloproteinases, *Lox*, *Tgm2*, and *Tgfb1* (*n* = 10). (**B** and **C**) Collagen deposition was assessed by the area of collagen in Masson’s trichrome staining (**B**) and hydroxyproline assay (**C**). *n* = 8–11, as indicated in the dot plots. (**D**) Elastin contents were measured in aortic samples, using biochemical assays (*n* = 8–11, as indicated in the dot plot). All data are presented as mean ± SEM. Two-way ANOVA with Tukey’s multiple comparisons were used for data analysis. *P < 0.05 vs. ISM-Cre, vehicle.

Next, we investigated the effect of RhoBTB1 on arterial stiffness contributors in the ECM. Interestingly, the mRNA levels of predominant collagen genes and elastin (*Eln*) were not significantly changed upon RhoBTB1 restoration (Figure 7A). Moreover, after *Rhobtb1* transgene activation, there were no clear trends to implicate changes in the expression of genes that regulate ECM secretion, degradation, or cross-linking, such as matrix metalloproteinases, tissue inhibitors of metalloproteinases, lysyl oxidase (*Lox*), transglutaminase 2 (*Tgm2*), or *Tgfb1* (Figure 7A). As observed using quantitative RT-PCR, Ang II downregulated *Tgfb1* and 1 of its target genes, *Ctgf*, but RhoBTB1 did not reverse this effect (Supplemental Figure 4).

We also measured the abundance of collagen using 2 separate assays: Masson’s trichrome staining of the same sections used for the wall measurement described earlier (Figure 7B) and a biochemical assay for hydroxyproline (Figure 7C). Quantification of both methods suggests that Ang II markedly increased collagen deposition in the aorta, whereas RhoBTB1 restoration was insufficient to reverse this deposition. Similarly, Ang II decreased elastin abundance in the aorta in both ISM-Cre and S-RhoBTB1 mice to a comparable level (Figure 7D).

Last, there was also no apparent difference between Ang II–treated S-RhoBTB1 and ISM-Cre mice in terms of advanced glycation end products, a pathological contributor to the cross-linking of the ECM (Supplemental Figure 5), or in the level of calcification as measured by alizarin red staining (Supplemental Figure 6).

**Figure 8:**
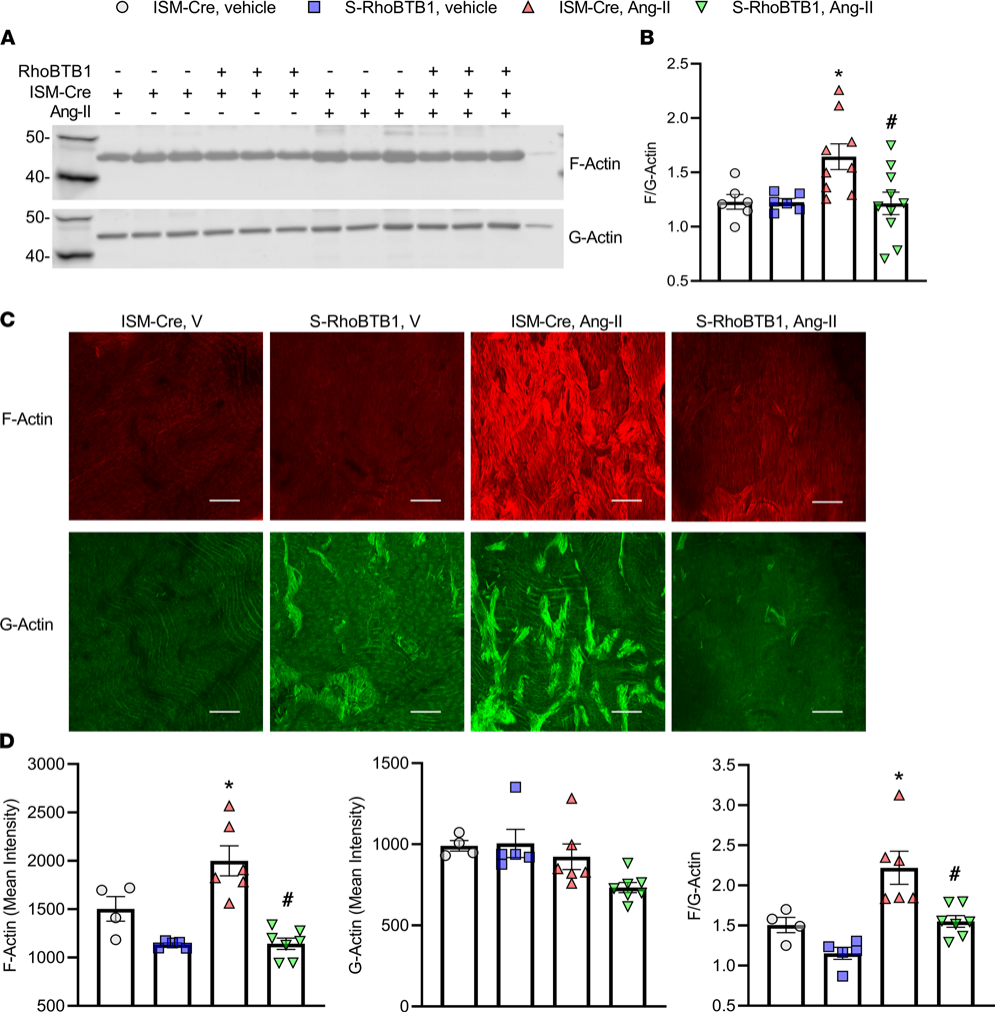
Actin polymerization. (**A** and **B**) F-actin and G-actin were separated by ultracentrifugation and assessed by Western blot (with 6–10 biological replicates, as indicated in the dot plot in **B**). The last lane was loaded with purified actin as a positive control. Molecular weight markers are as indicated in kilodaltons. (**B**) Densitometry was used to quantify F-actin and G-actin content in the Western blot. Actin polymerization was indicated by the ratio of F-actin to G-actin (*n* = 6–10, as indicated in the dot plot). One sample from the ISM-Cre vehicle group and another from the S-RhoBTB1 vehicle group were identified as outliers and were excluded using the Grubb’s outlier test. (**C**) Actin polymerization was confirmed using fluorescence staining in aorta (*n* = 4–7, as indicated in the dot plot in **D**). F-actin was stained with Alexa Fluor 594–phalloidin (red, upper panels) and G-actin was stained by Alexa Fluor 488–DNase I (green, lower panels). Scale bar: 100 μm. (**D**) MFI of the same volume of aortic samples was measured under the same optical configuration using confocal microscopy. Ratio of F-actin to G-actin was used to assess action polymerization. All data are presented as mean ± SEM (*n* = 4–7, as indicated in the dot plot). Two-way ANOVA with Tukey’s multiple comparisons were used for data analysis. *P < 0.05 vs. ISM-Cre, vehicle; ^#^*P* < 0.05 vs. ISM-Cre, Ang II.

To increase our mechanistic depth, we performed a meta-analysis to identify genes that were significantly upregulated or downregulated by Ang II and normalized upon RhoBTB1 restoration. Pathway enrichment analysis of this gene set identified significant changes in the cytoskeleton-related pathways (Supplemental Figure 7). This was of interest because there is emerging evidence suggesting that actin polymerization, the dynamic process of assembling globular actin (G-actin) monomers into filamentous actin (F-actin), in vascular SMCs plays a critical role in arterial stiffness (18–20). First, ultracentrifugation fractionation was used to physically separate F-actin from G-actin in aortic samples, which were resolved by Western blot (Figure 8A). The ratio between F- and G-actin was used as an indicator of actin polymerization. Interestingly, Ang II increased actin polymerization, whereas RhoBTB1 restoration reversed this effect (Figure 8B). Second, this observation was supported by quantification of fluorescence labeling of F-actin with phalloidin and of G-actin with deoxyribonuclease I (DNase I) (Figure 8C) in the aorta (21). Similar to the actin fractionation experiment, this staining revealed the increase in actin polymerization caused by Ang II was normalized upon RhoBTB1 restoration (Figure 8D).

**Figure 9:**
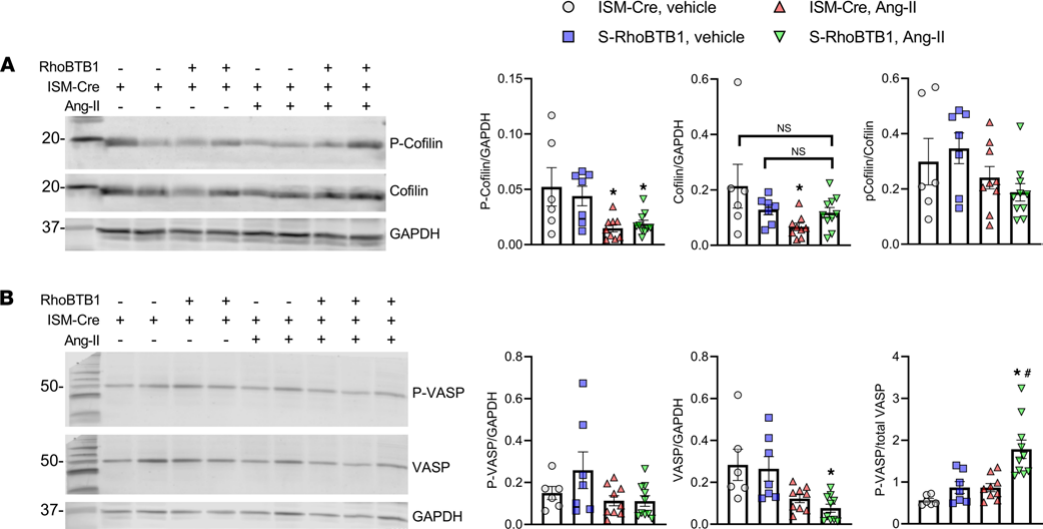
Cofilin and VASP. (**A**) Aortic P-cofilin and cofilin levels were detected by Western blotting (*n* = 6–10, as indicated in the dot plots). (**B**) P-VASP and total VASP levels were assessed by Western blot in aortic samples (*n* = 6–10, as indicated in the dot plots). These Western blots were performed with 6–10 biological replicates in 3 (cofilin) or 4 (VASP) blots for each probe. Measurements of all samples were reflected in the corresponding Western blot analysis panel. Molecular weight markers are as indicated in kilodaltons. All data are presented as mean ± SEM. Two-way ANOVA with Tukey’s multiple comparisons were used for data analysis. *P < 0.05 vs. ISM-Cre, vehicle. ^#^*P* < 0.05 vs. ISM-Cre, Ang II. NS, not significantly different from vehicle-treated samples.

Finally, we assessed the levels of cofilin and serine-3-phosphorylated cofilin (P-cofilin). Cofilin accelerates actin depolymerization, and the phosphorylated form is the inactivated form of cofilin (22). Interestingly, Ang II alone decreased the level of both cofilin and P-cofilin. Restoration of RhoBTB1 in Ang II–treated S-RhoBTB1 mice restored cofilin to baseline levels (Figure 9A). We also examined the level of vasodilator-stimulated phosphoprotein (VASP), because it is another prominent contributor to actin polymerization (23). Like cofilin, the Ser239-phosphorylated form of VASP (p-VASP) inhibits VASP activity. Ang II lowered the levels of both p-VASP and VASP in the aorta (Figure 9B). Restoration of RhoBTB1 increased the ratio of p-VASP compared with total VASP. Collectively, these observations are consistent with the finding that RhoBTB1 restoration reversed actin polymerization in Ang II–treated mice, further validating a role for RhoBTB1 in cytoskeletal organization.

## Discussion

In this study, we show that RhoBTB1 restoration in the vasculature attenuated established arterial stiffness but had no effect on BP and vascular function. Thus, RhoBTB1 efficiently and rapidly reversed established arterial stiffness even during the preservation of hypertension and vascular dysfunction. Thus, one of the important conclusions from this study is that there are mechanisms underlying the rapid reversal of arterial stiffness that can occur independently of BP and with the continuous presence of hypertension and vascular dysfunction. Interestingly, RhoBTB1 did not reverse many of the classical mechanisms considered when typically evaluating arterial stiffness, such as vascular hypertrophy and collagen deposition. Alternatively, RhoBTB1 regulated the state of actin polymerization in vascular SMCs, which appears to be a molecular function for RhoBTB1 in the vascular wall.

*Rhobtb1* is a transcriptional target of PPARγ. *PPARG* mutations cause hypertension in humans (24), and *RhoBTB1* has been associated with BP (12, 13). In mice, interference with PPARγ signaling in vascular SMCs results in hypertension, vascular dysfunction, and arterial stiffness; furthermore, *Rhobtb1* expression decreases concurrently with these phenotypes (14, 15). Restoration of RhoBTB1 by a PPARγ-independent mechanism reverses all these phenotypes in mice carrying dominant-negative PPARγ mutation (16). Activation of PPARγ by thiazolidinediones, high-affinity agonists for PPARγ, lowers arterial pressure and exerts vasoprotective effects in patients with type 2 diabetes enrolled in the PROactive trial of pioglitazone (25). Activation of PPARγ has been shown to lower BP in many preclinical models of hypertension (reviewed in ref. 11) and, importantly, improves arterial stiffness in models of diabetes (26). However, thiazolidinediones also have been reported to result in a number of adverse effects (reviewed in ref. 27). Thus, our focus has been on identifying and understanding the downstream targets of PPARγ, like *Rhobtb1*, to capitalize on the beneficial effects while minimizing adverse events. Here, we focused on the protective effects of RhoBTB1 in Ang II–induced hypertension not only because Ang II hypertension represents more systemic and intricate pathologies but also because Ang II decreases RhoBTB1 levels in the vasculature.

The downregulation of RhoBTB1 by Ang II may be the product of 2 known mechanisms acting simultaneously. First, it is well established that Ang II suppresses the activity of PPARγ, which could interfere with the transactivation of *Rhobtb1* by PPARγ in vascular SMCs (28). Second, it is notable that a miRNA, miR-31, which targets the 3′ UTR of *Rhobtb1* mRNA can be induced by Ang II in the aorta (29, 30). Perhaps, the downregulation of *Rhobtb1* by Ang II may be the product of both mechanisms acting simultaneously, which may be interpreted as attempts to maintain the structural integrity (e.g., stiffness) of arterial wall and prevent aneurysm or rupture under conditions of marked hypertension. Importantly, the *Rhobtb1* transgene used in this study is unlikely to be affected by miR-31, because the transgene only spans the coding region and excludes the 3′ UTR targeted by miR-31.

Notably, in the current model, in which Ang II hypertension was established prior to transgene activation (e.g., a reversal model), RhoBTB1 exhibited physiological roles and molecular functions that are distinct from those observed in our previous studies (discussed below). Here, restoring RhoBTB1 after Ang II hypertension was established had no pronounced effect in BP or vasodilation; instead, it rescued arterial stiffness. However, in hypertension caused by smooth muscle–specific, dominant-negative PPARγ mutation, RhoBTB1 reversed hypertension by improving vasodilation. Additionally, in a prevention protocol where *Rhobtb1* transgene was inducibly activated before Ang II administration, RhoBTB1 only partially prevented the pressor response to Ang II. This partial prevention of hypertension might occur because Ang II hypertension involves not only vascular SMCs but also a myriad of organs and various cell types (16). These markedly different phenotypes from the same beneficial gene, RhoBTB1, emphasize the importance of investigating a target under different pathological backgrounds and chronologic stages of disease. Importantly, the reversal protocol we used in this study is significant because it tests the therapeutic potential of RhoBTB1 by emulating a treatment regimen given to a patient after clinical diagnosis.

The cause and effect relationship between hypertension and arterial stiffness remains an ongoing debate (31, 32). It is common for arterial stiffness and high BP to be observed simultaneously, thus leading many to conclude the development of arterial stiffness depends on elevated BP. Here, we report an intriguing observation that restoring RhoBTB1 attenuated arterial stiffness without either lowering BP or improving vasodilation. One potential limitation of this study is the duration of the BP measurements, which were limited by the life span of the radiotelemeter battery and the duration of the osmotic minipumps. One could argue that a difference in BP might eventually develop outside the time scale of our protocol, but this idea is challenged by the complicated pathophysiology of Ang II hypertension and the multisystemic mechanisms by which Ang II causes hypertension. Indeed, Ang II contributes to hypertension not only by increasing arterial stiffness but also through many other pathways, such as increasing oxidative stress, inflammation, sodium retention, and sympathetic outflow. Restoring 1 gene in 1 cell type might simply be insufficient to counteract all these mechanisms. The multitude of Ang II–dependent mechanisms may also be reflected in the extent of variation noted in the transcriptome analysis. Nonetheless, our results show that reduced arterial stiffness precedes detectable changes in BP or other dysfunctions caused by Ang II. Thus, reversal of Ang II–induced arterial stiffness does not appear to be a secondary effect of interfering with other direct effects caused by Ang II.

Pulse pressure has been used as a clinical parameter to evaluate arterial stiffness. However, we did not observe any decrease in pulse pressure despite the attenuation in arterial stiffness. There are 2 possible reasons for this observation. First, unlike aging, diabetes, or isolated systolic hypertension, Ang II elevates both systolic and diastolic BP by acting on multiple organs. For instance, Ang II impairs vasodilation, promotes vasoconstriction, increases myogenic tone, increases sympathetic activity, and promotes antinatriuresis. Second, pulse pressure can be affected by local hemodynamics, such as lumen diameter and aortic flow, as well as wave reflection and ventricular interaction (32). Indeed, the dissociation between arterial stiffness and pulse pressure has been observed in another mouse model (19).

Sex difference has been observed in many cardiovascular diseases. However, the present study was limited to male mice because of our use of the *Myh11*-Cre model (i.e., the ISM-Cre mice), whose Cre insertion is in the Y chromosome (33, 34). This strain was selected because it had the best reported cell specificity (35). It would be appropriate in future studies to extend our understanding of RhoBTB1 in female mice and investigate any potential sex differences.

Mechanistically, we previously reported that RhoBTB1 inhibits PDE5 activity, thus promoting the NO–cyclic cGMP (NO-cGMP) pathway (16). Because RhoBTB1 restoration did not improve vasodilation responses to either ACh or sodium nitroprusside in this model of Ang II hypertension, it is more likely that the NO-cGMP pathway did not contribute to the decreased arterial stiffness observed in this study. This suggests that RhoBTB1 might have other targets in the vasculature. One such target might be Rho-associated protein kinase1 (ROCK1), a downstream target of RhoA that regulates multiple biological processes, such as cell contraction and actin polymerization (36). In particular, RhoBTB1 was reported to co-immunoprecipitate with ROCK1 in COS7 cells and to inhibit cancer cell invasion by an unclear mechanism (37). It was proposed that ROCK1 phosphorylates RhoBTB1, which prevents RhoBTB1’s association with Cullin-3, thus blunting the turnover of RhoBTB1-binding partners by the Cullin-3 ubiquitin ligase complex. Interestingly, Ang II is known to increase RhoA/ROCK signaling (38). Indeed, we observed increased contraction to agonists that act through RhoA/ROCK–dependent mechanisms in Ang II–treated ISM-Cre and S-RhoBTB1 mice. Thus, RhoBTB1 reversed arterial stiffness despite increased contractile activity, suggesting the reversal of arterial stiffness may not depend on altering ROCK activity. Nonetheless, the relationship among RhoBTB1, ROCK, and Cullin-3 activities in vascular SMC regulation merits further investigation.

Classical Rho GTPases, such as RhoA, Rac1, and CDC42, function as molecular switches by cycling between GTP/GDP–bound conformations and are well known for inducing actin polymerization (39). RhoBTB1 is an atypical Rho GTPase with a markedly different molecular structure. First, the GTPase domain of RhoBTB1 contains multiple deviations that may impair its GTPase activity, and there remains a lack of direct evidence on whether RhoBTB1 binds to GTP, GEFs, or GAPs. Second, RhoBTB1 carries many additional domains, including a proline-rich domain, 2 BTB domains, and a unique C-terminus. Thus, previous evidence suggests that RhoBTB1 functions distinctly from classical Rho GTPases (40). However, our observation that RhoBTB1 downregulates actin polymerization in vasculature challenges this idea. RhoBTB1 might thus resemble other members of the atypical Rho GTPase family, including RhoH and Rnds/RhoE. Interestingly, both RhoH and Rnds carry deviations in their GTPase domain, inhibit RhoA and Rac1, and antagonize actin polymerization (41, 42). Under the context of our previous finding that RhoBTB1 serves as a ubiquitin ligase substrate adaptor through its BTB domains (16), we hypothesize that RhoBTB1 is a multifunctional protein that may act through several of its domains to affect vascular regulation. Clearly, more studies are needed to identify other targets of RhoBTB1, which domains they interact with, and the physiological outcomes of those interactions.

## Methods

### Experimental animals.

S-RhoBTB1 mice expressing a Tx-inducible, smooth muscle–specific *Rhobtb1* transgene were generated by breeding RhoBTB1^IND^ mice with ISM-Cre mice, as previously described (16). Briefly, RhoBTB1^IND^ mice carry the transgene composed of the following: (a) CAG promoter, (b) LoxP-STOP-LoxP sequence, (c) full-length mouse *Rhobtb1*, and (d) IRES-tdTomato sequence. ISM-Cre mice express Tx-inducible Cre recombinase under the control of smooth muscle myosin heavy chain promoter (019079, Jackson Laboratory). All experimental animals were maintained by backcross breeding (>6 generations) to C57BL/6J. Male mice were used in this study because the Cre recombinase–containing BAC gene was inserted in the Y chromosome of ISM-Cre mice.

S-RhoBTB1 mice and ISM-Cre littermates at 3 to 5 months of age received a 6-week infusion of either vehicle (NaCl–acetic acid buffer) or Ang II (A9525, MilliporeSigma) via osmotic minipumps (model 2006, Alzet). Two weeks after pump implantation, the transgene was induced by 5 consecutive, daily, i.p. injections of Tx (MilliporeSigma) (75 mg/kg). Tissue harvest was performed 2 weeks after the completion of Tx injections.

Although most of the data were generated at the Medical College of Wisconsin (MCW), some of the BP measurements (which were replicated at MCW) were obtained at the University of Iowa. All procedures were approved by the IACUC at the MCW and the University of Iowa following the NIH *Guide for the Care and Use of Laboratory Animals* (National Academies Press, 2011).

### Immunostaining.

Aorta were fixed under physiological pressure using 10% neutral buffered formalin. Paraffin-embedded aorta cross sections were subjected to immunofluorescence staining or IHC staining following the standardized protocols on the automated staining platform (BOND RX, Leica). For the immunofluorescence staining, primary Abs against endothelium marker CD31 (1:100; AF3628, R&D Systems) and the transgene reporter tdTomato (1:200; 127897, GeneTex) were incubated with the samples at 4°C overnight. For the IHC staining, a rabbit polyclonal Ab against advanced glycation end products (1:200; ab23722, Abcam) was used.

### BP measurements.

Radiotelemetry was used to record real-time BP as previously reported (16). After implantation of radiotelemeters (PA-C10, DSI) in the left common carotid artery, mice were given 10 days to recover before BP recordings were started. Ketamine and xylazine were used as anesthesia while meloxicam was used as analgesia postsurgery. Systolic, diastolic, and mean arterial BPs and heart rate were continuously recorded for 10 minutes every hour during the entire protocol. Pulse pressure was calculated by subtracting the diastolic BP from systolic BP. Baseline BP was recorded for 7 days prior to Ang II pump implantation. Hourly and daily averages of BP and heart rate were calculated and exported by the Ponemah software (version 6.50; Data Sciences International).

### Arterial stiffness and compliance.

PWV measured by the Doppler Flow Velocity System (Indus) was used to evaluate arterial stiffness, as previously reported (16). Mice were anesthetized with isoflurane (2% oxygen at 2 L/min) and secured on a 37°C heating pad in a supine position. Heart rate was monitored by ECG electrodes on the heating pad and maintained between 450 and 500 bpm. Two 20 MHz ultrasound probes were positioned at the descending and abdominal aorta to record the arrival time of pulse at each position. Transit time, the amount of time for the pulse to travel between the 2 positions, was averaged over 5 cycles. PWV was calculated by dividing the distance between the descending and abdominal aorta by the transit time.

Pressure-diameter curves were used to measure arterial compliance. Aortic segments were mounted on the force chamber of a pressure myograph (Pressure Interface, Danish Myo Technology) and incubated in calcium-free Krebs buffer to eliminate active vascular reactivity. Under a no-flow condition, intraluminal pressure was increased from 0 mmHg to 200 mmHg with increments of 25 mmHg. The outer and inner diameters were recorded by video microscopy (Zeiss Vert.A1). Changes in outer diameter were used to plot the pressure-diameter curves.

The stress-strain curves were calculated as previously described (43): circumferential stress = (intraluminal pressure × inner diameter)/(2 × wall thickness); strain = (D – D0)/D0, where D is the pial outer diameter and D0 is the original outer diameter. The outer diameter at 5 mmHg was used instead of 0 mmHg to avoid the arteries collapsing.

### Vascular function.

Wire myography was used to study the vascular reactivity in carotid and mesenteric arteries, as previously described (44). Briefly, mice were euthanized by pentobarbital (150 mg/kg; Fatal-Plus, Vortech Pharmaceuticals), and carotid artery rings were carefully dissected and then mounted on a force transducer. After 30 minutes of equilibration at 0.25 g in 37°C Krebs buffer (NaCl, 118 mM; KCl, 4.7 mM; MgSO_4_, 1.2 mM; KH_2_PO_4_ monobasic, 1.2 mM; NaHCO_3_, 25.0 mM; CaCl_2_, 2.5 mM; and glucose, 11.0 mM) with 95% O_2_ and 5% CO_2_ bubbling, carotid artery rings were treated with KCl (10 mM, 30 mM, 100 mM), serotonin (10^–8^ to 10^–5^ M), or endothelin-1 (10^–10^ to 3 × 10^–8^ M) to study the vasoconstriction response. For vasodilation responses, carotid artery rings were preconstricted with U46619 (16450, Cayman Chemical) to 40% to approximately 70% of maximum contraction, then treated with ACh (10^–9^ to 3 × 10^–5^ M) or sodium nitroprusside (10^–10^ to 3 × 10^–5^ M). Tension in the carotid rings was collected using PowerLab (ML846) and analyzed by LabChart software (version 8).

Similarly, second-order mesenteric arteries (2 mm long) were mounted on 2 tungsten wires (25 μm diameter) on a wire myograph (DMT620M, Danish Myo Technology). Preload tension was set to IC_90_ and applied to all mesenteric rings, as previously described (45). Mesenteric rings were submaximally contracted with U46619 prior to cumulative concentration-response curves, as described above. Data are reported as percentage relaxation or maximal force generation (in millinewtons).

### Aortic quantitative PCR.

Aortas were homogenized using a bead mill (1.4 mm ceramic beads; 15-340-153, Fisherbrand, Thermo Fisher Scientific). Aortic RNA was isolated with the Purelink RNA Mini Kit (12183020; Invitrogen), following the manufacturer’s instruction. For each sample, 400 ng of aortic mRNA was reverse-transcribed into cDNA using the High-Capacity cDNA Reverse Transcription Kit (Applied Biosystems). We performed quantitative PCR using the StepOnePlus Real-Time PCR System (Applied Biosystems). The following TaqMan probes were used to measure relative gene expression levels: (a) *Rhobtb1* (Mm01143659_m1), customized TaqMan probes for *Rhobtb1* exon6 (which encodes most RhoBTB1 protein, from the GTPase domain to the BTB domains); (b) *Klf4* (Mm00516104_m1); (c) *Tgfb1* (Mm01178820_m1); (d) *Ctgf* (Mm01192933_g1); and (e) *18s* (Mm04277571).

### RNA-Seq.

Total aortic RNA was isolated using the same approach as mentioned in the previous paragraph. RNA was quantified using fluorometric methods (Qubit, Invitrogen), and quality was assessed with Fragment Analysis (Agilent). Library preparation was initiated with 500 ng of RNA and completed following manufacturer’s recommendations for the TruSeq Stranded mRNA Kit (Illumina). Final libraries were quantified with quantitative PCR (Kapa Library Quantification Kit, Kapa Biosystems), and fragment size was observed with high-sensitivity DNA fragment analysis (Agilent). Paired-end sequencing was completed at the Genomic Sciences and Precision Medicine Center (at MCW) using the NovaSeq 6000 at 2 × 100-bp reads, targeting 50 million reads per sample. Data were trimmed and quality was assessed with FastQC and RSeQC. Reads were aligned to the mouse reference Gencode vM23 (based on Ensembl v98) using the STAR aligner and MAPR-Seq workflow version 3.0 (46). Standardized counts per million (CPM) of global genes were used for the PCA to visualize transcriptome variation among groups. Differential gene expression analysis was performed using the R (version 4.0.3) package *edgeR* (47). Genes with FDR of no more than 0.05 and log_2_ fold change at least 1 or less than or equal to –1 were considered differentially expressed (DE) genes. For the genes displayed in heatmaps, standardized log_2_CPM was plotted to indicate expression level. Data were deposited (NCBI Gene Expression Omnibus accession series GSE189003).

### Meta-analysis.

To identify the genes that were significantly upregulated by Ang II and decreased upon RhoBTB1 restoration or vice versa, we performed a meta-analysis using the “maxP.OC” method in the R package *metaOmics* (48). This method takes the maximum of 1-sided *P* values from different comparisons (with a 1-sided correction) as the test statistic and derives meta-analyzed *P* values. Multiple-testing correction is controlled by FDR using the Benjamini-Hochberg procedure (49). Genes with FDR < 0.05 were considered significant in multiple comparisons with concordant signs of effects.

Pathway enrichment analysis was performed with the significant genes identified in the meta-analysis, using the Multi Ontology Enrichment Tool (MOET) in the Rat Genome Database developed by MCW (https://rgd.mcw.edu/rgdweb/enrichment/start.html) (50). Given a gene list, MOET identifies significantly overrepresented ontology terms using a hypergeometric test and provides nominal and Bonferroni-adjusted *P* values. Terms in the cellular component ontology with adjusted *P* < 0.05 were shown in the bubble plot (see Supplemental Figure 6). Rich factor was calculated by dividing the number of the significant genes by the total number of genes in each term.

### Western blot.

Protein lysates were extracted from mice aorta using a lysis buffer (50 mM Tris HCl, 1% wt/vol sodium deoxycholic acid, 1% wt/vol NP-40, 0.1% wt/vol SDS, 0.1 mM EDTA, and 0.1 mM EGTA) supplemented with a protease inhibitor cocktail (11836170001, Roche) and phosphatase inhibitor (4906845001, Roche). Protein concentration was measured (5000112, Bio-Rad) and 30 μg of protein lysate was loaded into each lane. Protein lysates were separated using SDS-PAGE (5% to 12%) and transferred on PVDF membranes (IPVH20200, MilliporeSigma). After 1.5 hours of blocking in 5% nonfat milk, the membranes were incubated with primary Abs at 4°C overnight. For the tdTomato experiment, proteins were probed by HRP-conjugated secondary Abs (NXA931V, NA934V, Cytiva). For actin and cofilin assays, the LI-COR Odyssey CLx and corresponding fluorescently labeled Abs (926-32211, 926-68070, LI-COR) were used. The following primary Abs were used to detect target proteins: tdTomato (GTX127897, GeneTex), actin (AAN02-S, Cytoskeleton, Inc.), P-cofilin (3313, Cell Signaling Technology), cofilin (5175, Cell Signaling Technology), p-VASP (3114, Cell Signaling Technology), VASP (3132, Cell Signaling Technology), and GAPDH (sc-32233, Santa Cruz Biotechnology). See complete unedited blots in the supplemental material.

### Collagen and elastin deposition.

Collagen deposition was measured using the hydroxyproline assay (16, 51). In brief, aortas were digested in 0.1N NaOH solution at 90°C for 16 hours, and both the insoluble and soluble fractions were hydrolyzed into amino acids in 6N HCl (SA56-500, Thermo Fisher Scientific) overnight. The soluble fraction was reconstituted in sodium acetate–trisodium citrate–isopropanol buffer, oxidized with chloramine-T trihydrate (402869, MilliporeSigma) at room temperature for 5 minutes, and then incubated with Erlich’s reagent [4-(dimethylamino) benzaldehyde, D2004, MilliporeSigma; 70% perchloric acid, Spectrum P1025] at 60°C for 30 minutes before OD at 558 nm was measured. Collagen content was normalized by total protein content (measured in nanograms per microgram). Elastin was measured by the ratio of the protein content in the insoluble compartment obtained from the NaOH digestion to the total protein content.

### Actin polymerization.

Actin polymerization was measured using G-Actin/F-Actin In Vivo Assay Biochem Kit (BK037, Cytoskeleton) with minor modifications. Briefly, an equivalent length of frozen aorta was ground into powder and mixed with 800 μL lysis buffer containing proprietary F-actin stabilization reagents provided by the kit. After incubation at 37°C for 20 minutes, lysates were centrifuged (Optima TLX/tube 357448, Beckman Coulter) at 112,000*g* (50,000 rpm for rotor TLA-55) for 1 hour at 37°C. The supernatant was collected to detect G-actin, and the precipitant was further incubated with 120 μL of F-actin depolymerization buffer (8 M urea) on ice for 1 hour with vigorous vortexing every 15 minutes. Finally, the solubilized F-actin fraction was collected by centrifugation at 2300*g* for 5 minutes at 4°C. To determine the F-actin to G-actin ratio, an equal volume of F-actin and G-actin lysates were subjected to Western blotting using primary Ab against mouse actin (AAN02, Cytoskeleton) and secondary goat anti-mouse Ab (1:10,000; IRDye 800CW goat anti-mouse 827-08364, LI-COR). Band densitometry was performed using the LI-COR imaging system (Odyssey CLx).

Fluorescence labeling of F-actin with phalloidin and G-actin with DNase I was also used to assess actin polymerization. Mice were perfused first with PBS, then with 4% paraformaldehyde at a flow rate of 38 mL/min for vehicle-treated mice and 50 mL/min for Ang II–treated mice. Such flow rates were adapted to mimic the average physiological BP, because actin polymerization is a dynamic process that is sensitive to mechanical stress. The thoracic aorta was dissected, and the adventitia was removed before the staining to ensure vascular SMCs access to the probes and avoid autofluorescence. Tissue was permeabilized with 0.1% Triton X-100 for 10 minutes, followed by 3 washes with PBS, and then stained with Alexa Fluor 488–DNase I (D12371, Molecular Probes) and Alexa Fluor 594–phalloidin (A12381, Invitrogen) for 40 minutes at room temperature. Subsequently, the tissue was rinsed and mounted without sectioning using mounting medium containing DAPI. Images (original magnification, ×20) were captured using a Nikon A1 laser scanning microscope under the same thickness, laser power, and gain. MFI was obtained by projecting all layers in the *Z*-stack to the same plane, and the average of 3 different areas from the same sample was calculated and plotted.

### Statistics.

All data are presented as mean ± SEM. Statistical analysis was performed using GraphPad Prism 9.0 (except for RNA-Seq DE analysis and meta-analysis, for which we used the statistical software R). *P* < 0.05 was considered statistically significant. Two-way ANOVA was used and repeated measurement, Tukey’s multiple comparisons, or Bonferroni multiple comparisons was applied when appropriate. Outliers were identified by Grubb’s test and reported in applicable figure legends.

### Study approval.

All mice were housed in the University of Iowa Transgenic Facility or MCW Biomedical Research Center Vivarium. Animal care was carried out per the guidelines and standards recommended by NIH. All experiments and procedures were approved by the IACUC of University of Iowa and MCW.

## Author contributions

SF was responsible for the experimental design, data collection, data analysis, and manuscript composition. JW designed experimental protocols (pulse wave velocity, wire, pressure myography, and the hydroxyproline assay) and performed some of the telemetry surgeries. JJR performed the mesenteric artery wire myography and assisted with sample collection. KTL collected samples for wire myography and composed the study approval paragraph. GK assisted with protocol development and performance of the actin experiments. AMW collected the samples for the histological studies. DTB performed telemetry surgeries and sample collections. SDS performed wire myography studies. PCMV collected samples for the actin polymerization histological studies. KKW was responsible for all the animal breeding and care. AJM directed the library preparation, sequencing of the bulk RNA, and bioinformatic analysis to differential gene expression. BQT and CWL performed RNA-Seq data analysis and visualization. FWQ provided extensive discussion about and mentoring for the project. CDS provided funding, supervised the design, reviewed data for all the experiments in this project, and critically reviewed and revised the manuscript.

## Supplementary Material

Supplemental data

Supplemental table 1

## Version 1 03/31/2022

In-Press Preview

## Version 2 05/09/2022

Electronic publication

## References

[1] Virani SS (2020). Heart Disease and Stroke Statistics-2020 update: a report from the American Heart Association. Circulation.

[2] Dunbar SB (2018). Projected Costs of Informal Caregiving for Cardiovascular Disease: 2015 to 2035: a policy statement from the American Heart Association. Circulation.

[3] Hessami A (2021). Cardiovascular diseases burden in COVID-19: systematic review and meta-analysis. Am J Emerg Med.

[4] Laurent S (2001). Aortic stiffness is an independent predictor of all-cause and cardiovascular mortality in hypertensive patients. Hypertension.

[5] Harvey A (2016). Vascular fibrosis in aging and hypertension: molecular mechanisms and clinical implications. Can J Cardiol.

[6] Boutouyrie P (2021). Arterial stiffness and cardiovascular risk in hypertension. Circ Res.

[7] Zieman SJ (2005). Mechanisms, pathophysiology, and therapy of arterial stiffness. Arterioscler Thromb Vasc Biol.

[8] Wagenseil JE, Mecham RP (2009). Vascular extracellular matrix and arterial mechanics. Physiol Rev.

[9] Lacolley P (2017). Vascular smooth muscle cells and arterial stiffening: relevance in development, aging, and disease. Physiol Rev.

[10] Durham AL (2018). Role of smooth muscle cells in vascular calcification: implications in atherosclerosis and arterial stiffness. Cardiovasc Res.

[11] Fang S (2021). Role of the peroxisome proliferator activated receptors in hypertension. Circ Res.

[12] Newton-Cheh C (2009). Genome-wide association study identifies eight loci associated with blood pressure. Nat Genet.

[13] Evangelou E (2018). Genetic analysis of over 1 million people identifies 535 new loci associated with blood pressure traits. Nat Genet.

[14] Pelham CJ (2012). Cullin-3 regulates vascular smooth muscle function and arterial blood pressure via PPARγ and RhoA/Rho-kinase. Cell Metab.

[15] Halabi CM (2008). Interference with PPAR gamma function in smooth muscle causes vascular dysfunction and hypertension. Cell Metab.

[16] Mukohda M (2019). RhoBTB1 protects against hypertension and arterial stiffness by restraining phosphodiesterase 5 activity. J Clin Invest.

[17] Miano JM (2010). Vascular smooth muscle cell differentiation-2010. J Biomed Res.

[18] Zhou N (2017). Rho kinase regulates aortic vascular smooth muscle cell stiffness via actin/SRF/myocardin in hypertension. Cell Physiol Biochem.

[19] Valisno JAC (2021). BCL11B regulates arterial stiffness and related target organ damage. Circ Res.

[20] Qiu H (2010). Short communication: vascular smooth muscle cell stiffness as a mechanism for increased aortic stiffness with aging. Circ Res.

[21] Hitchcock SE (1980). Actin deoxyroboncuclease I interaction. Depolymerization and nucleotide exchange. J Biol Chem.

[22] Coumans JVF (2018). Cofilin and profilin: partners in cancer aggressiveness. Biophys Rev.

[23] Benz PM (2009). Differential VASP phosphorylation controls remodeling of the actin cytoskeleton. J Cell Sci.

[24] Barroso I (1999). Dominant negative mutations in human PPARgamma associated with severe insulin resistance, diabetes mellitus and hypertension. Nature.

[25] Dormandy JA (2005). Secondary prevention of macrovascular events in patients with type 2 diabetes in the PROactive study (PROspective pioglitAzone clinical trial in macrovascular events): a randomised controlled trial. Lancet.

[26] Afzal S (2021). Peroxisome proliferator-activated receptor agonist (pioglitazone) with exogenous adiponectin ameliorates arterial stiffness and oxidative stress in diabetic Wistar Kyoto rats. Eur J Pharmacol.

[27] Singh S (2007). Long-term risk of cardiovascular events with rosiglitazone: a meta-analysis. JAMA.

[28] Alexis JD (2009). Bcr kinase activation by angiotensin II inhibits peroxisome-proliferator-activated receptor gamma transcriptional activity in vascular smooth muscle cells. Circ Res.

[29] Xiao J (2017). miR-31a-5p promotes postnatal cardiomyocyte proliferation by targeting RhoBTB1. Exp Mol Med.

[30] Li X (2019). MicroRNA-31 regulates immunosuppression in Ang II (angiotensin II)-induced hypertension by targeting Ppp6C (protein phosphatase 6c). Hypertension.

[31] Oh YS (2017). A special report on the NHLBI initiative to study cellular and molecular mechanisms of arterial stiffness and its association with hypertension. Circ Res.

[32] Mitchell GF (2014). Arterial stiffness and hypertension: chicken or egg?. Hypertension.

[33] Wirth A (2008). G12-G13-LARG-mediated signaling in vascular smooth muscle is required for salt-induced hypertension. Nat Med.

[34] Rautureau Y (2017). Generation of a mouse model with smooth muscle cell specific loss of the expression of PPARγ. Methods Mol Biol.

[35] Chakraborty R (2019). Promoters to study vascular smooth muscle. Arterioscler Thromb Vasc Biol.

[36] Loirand G (2006). Rho kinases in cardiovascular physiology and pathophysiology. Circ Res.

[37] Haga RB (2019). RhoBTB1 interacts with ROCKs and inhibits invasion. Biochem J.

[38] Hilgers RH (2007). Increased PDZ-RhoGEF/RhoA/Rho kinase signaling in small mesenteric arteries of angiotensin II-induced hypertensive rats. J Hypertens.

[39] Jaffe AB, Hall A (2005). Rho GTPases: biochemistry and biology. Annu Rev Cell Dev Biol.

[40] Aspenstrom P (2004). Rho GTPases have diverse effects on the organization of the actin filament system. Biochem J.

[41] Wennerberg K (2003). Rnd proteins function as RhoA antagonists by activating p190 RhoGAP. Curr Biol.

[42] Chae HD (2008). Cross-talk between RhoH and Rac1 in regulation of actin cytoskeleton and chemotaxis of hematopoietic progenitor cells. Blood.

[43] Hajdu MA (1990). Effects of aging on mechanics and composition of cerebral arterioles in rats. Circ Res.

[44] Wu J (2021). Failure to vasodilate in response to salt loading blunts renal blood flow and causes salt-sensitive hypertension. Cardiovasc Res.

[45] Reho JJ (2019). Smooth muscle cell-specific disruption of the BBSome causes vascular dysfunction. Hypertension.

[46] Kalari KR (2014). MAP-RSeq: Mayo analysis pipeline for RNA sequencing. BMC Bioinformatics.

[47] Robinson MD (2010). edgeR: a Bioconductor package for differential expression analysis of digital gene expression data. Bioinformatics.

[48] Ma T (2019). MetaOmics: analysis pipeline and browser-based software suite for transcriptomic meta-analysis. Bioinformatics.

[49] Benjamini Y, Hochberg Y (1995). Controlling the false discovery rate: a practical and powerful approach to multiple testing. J R Stat Soc Series B Stat Methodol.

[50] Kaldunski ML (2022). The Rat Genome Database (RGD) facilitates genomic and phenotypic data integration across multiple species for biomedical research. Mamm Genome.

[51] Cissell DD (2017). A modified hydroxyproline assay based on hydrochloric acid in Ehrlich’s solution accurately measures tissue collagen content. Tissue Eng Part C Methods.

