# Supplementary material for: RhoBTB1 reverses established arterial stiffness in angiotensin II–induced hypertension by promoting actin depolymerization
Source: JCI Insight. 2022 May 9;7(9):e158043. doi: 10.1172/jci.insight.158043 (PMC9090250; doi:10.1172/jci.insight.158043)
Supplement: Supplemental data [file jciinsight-7-158043-s165.pdf]

Supplemental Figures

RhoBTB1 Reverses Established Arterial Stiffness in Angiotensin-II Hypertension by Promoting Actin Depolymerization

Shi Fang[1, 2], Jing Wu[1], John J. Reho[1*], Ko-Ting Lu[1*], Daniel T. Brozoski[1*], Gaurav Kumar[1*], Alec Werthman[1], Sebastiao Donato Silva Jr[1], Patricia C. Muskus Veitia[1], Kelsey K. Wackman[1], Angela J. Mathison[3,4], Bi Qing Teng[5], Chien-Wei Lin[5], Fredrick W. Quelle[2], Curt D. Sigmund[1]

[1]Department of Physiology and Cardiovascular Center, Medical College of Wisconsin, Milwaukee, WI 53226, USA

[2]Department of Neuroscience and Pharmacology, Carver College of Medicine, University of Iowa, Iowa City, IA 52242, USA

[3]Genomic Sciences and Precision Medicine Center, Medical College of Wisconsin, Milwaukee, WI 53226, USA

[4]Division of Research, Department of Surgery, Medical College of Wisconsin, Milwaukee, WI 53226, USA

[5]Division of Biostatistics, Medical College of Wisconsin, Milwaukee, WI 53226, USA

* Contributed equally to the paper.

Conflict of interests: see conflicts on Page 30

Corresponding Author:

Curt D. Sigmund, Ph.D.
Department of Physiology
Medical College of Wisconsin
8701 Watertown Plank Road
Milwaukee, WI 53226-0509
414-955-8277
csigmund@mcw.edu

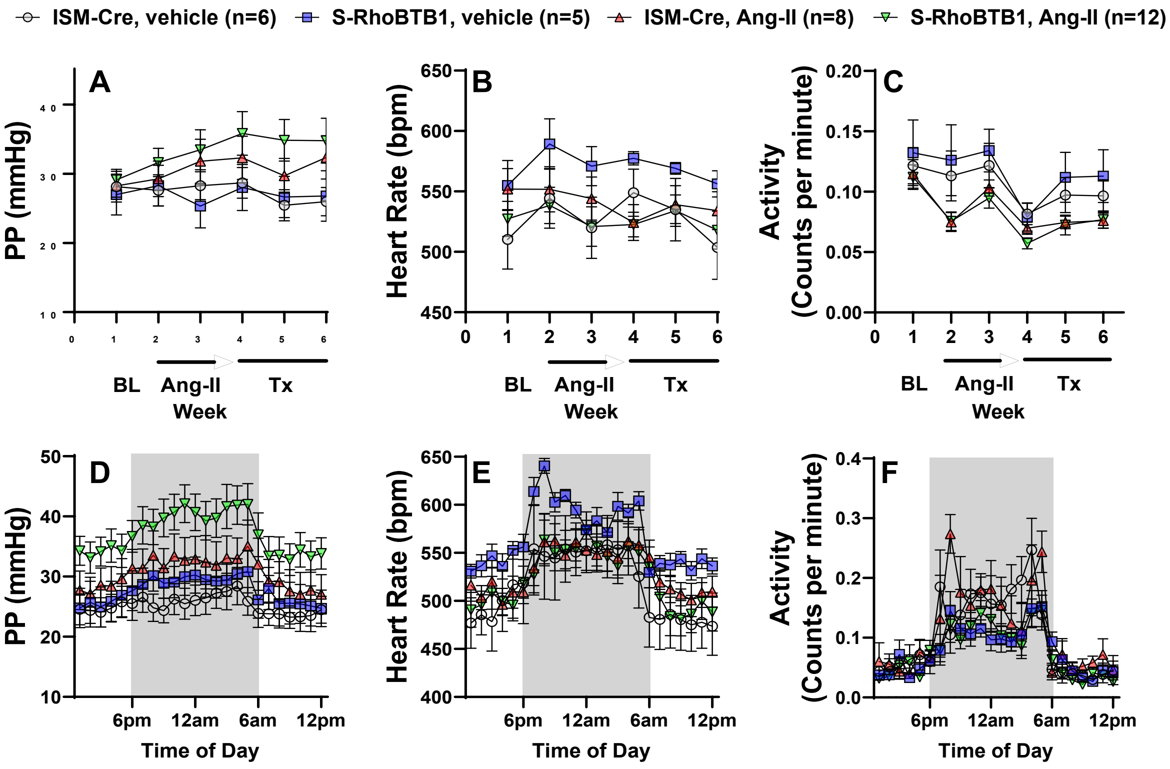

Supplemental Figure 1. Blood Pressure

Blood pressure was continuously measured by radiotelemetry (n=5-12). Pulse pressure (PP, **A**), heart rate **(B)**, and activity **(C)** during the entire protocol were presented as the weekly average. Two weeks after the transgene activation, hourly PP **(D)**, heart rate **(E)**, and activity **(F)** were calculated by 7-day average. All data are presented as a mean ± SEM. Two-way ANOVA and Tukey's multiple comparisons were used for data analysis at each time point. All data are presented as a mean ± SEM. * P <0.05 vs. ISM-Cre, vehicle.

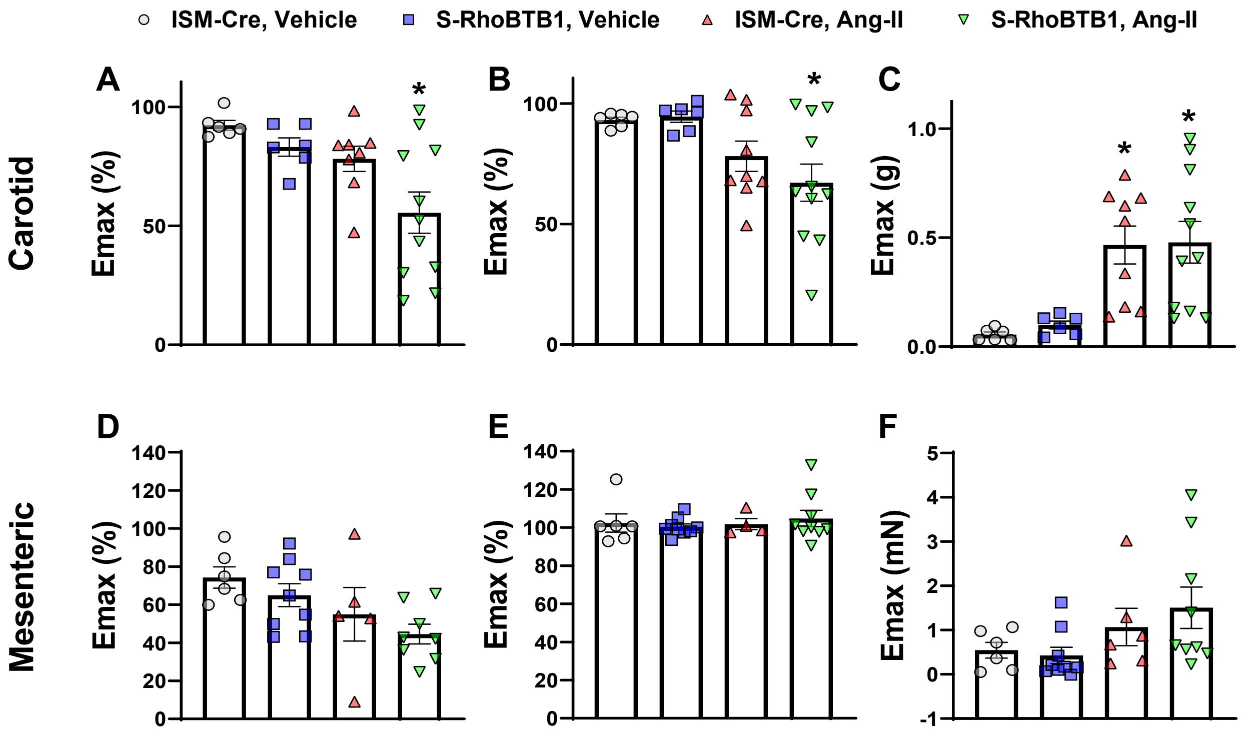

Supplemental Figure 2. Vascular Reactivity

Carotid arteries (n=6-11) **(A-C)** and mesenteric arteries (n=5-9) **(D-F)** were studied using wire myography. Maximum effect (Emax) of endothelial-mediated vasodilation response to acetylcholine (ACh) in carotid arteries **(A)** and mesenteric arteries **(D)**. Emax of endothelial-independent vasodilation response to sodium nitroprusside (SNP) in carotid arteries **(B)** and mesenteric arteries **(E)**. Emax of serotonin (5-HT) mediated vasoconstriction in carotid arteries **(C)** and mesenteric arteries **(F)**. All data are presented as mean ± SEM. * P <0.05 vs. ISM-Cre, vehicle. Two-way ANOVA and Tukey's multiple comparisons were used for statical analysis.

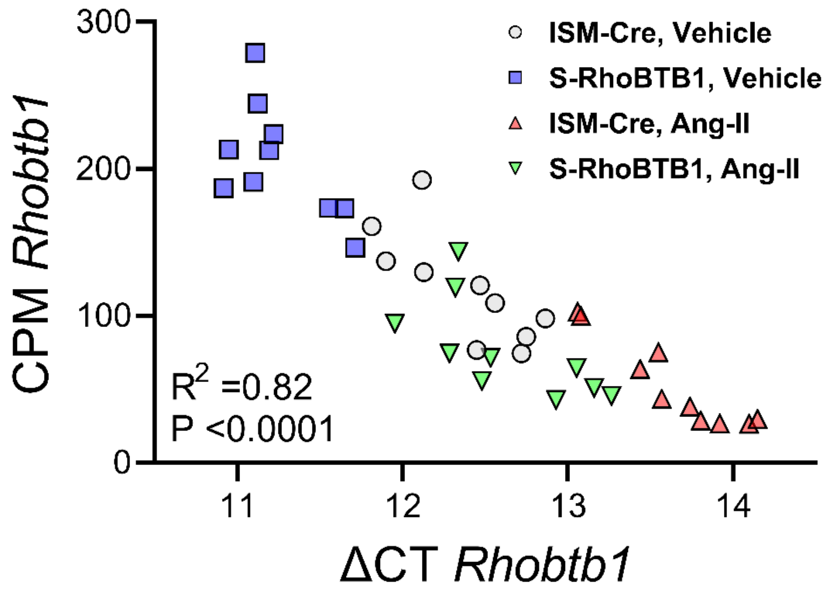

Supplemental Figure 3. Correlation between RNA-seq and RT-qPCR

To determine whether RNA-seq predicts the expression level of a gene, RhoBTB1 mRNA levels measured by both RNA-seq (counts per million, CPM) and RT-qPCR (ΔCT) were plotted on the same graph. Each data point indicates the CPM and ΔCT from the same sample (n=10 per group). Linear regression was performed with ungrouped data with all samples included (n=40).

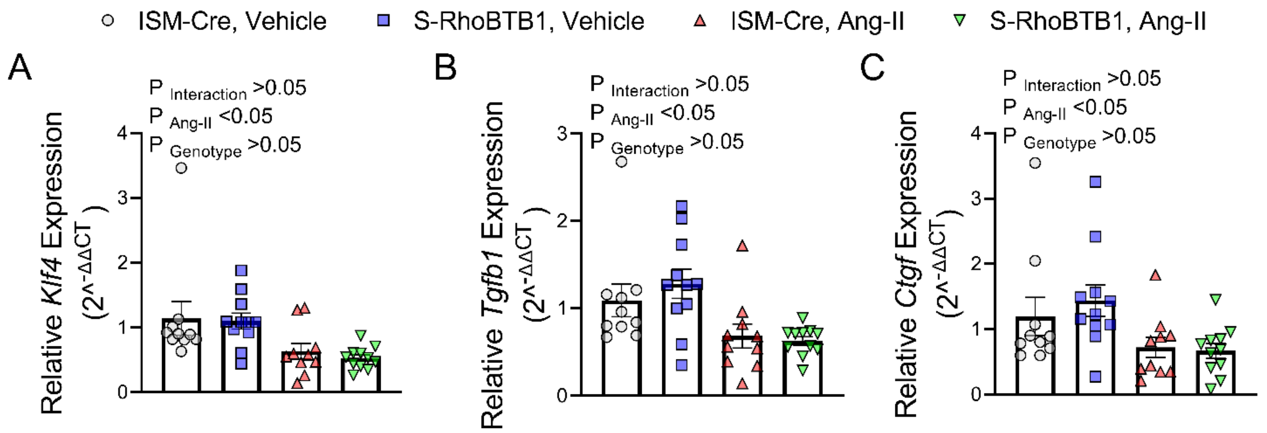

Supplemental Figure 4. Expression of KLF4, TGFB, and CTGF

Expression levels of three prominent genes that regulate vascular SMC differentiation (*Klf4*) and extracellular matrix production (*Tgfb1*, *Ctgf*) were measured in aorta lysates by RT-qPCR (n=10-11). All data are presented as mean ± SEM. Two-way ANOVA and Tukey's multiple comparisons were used for statical analysis.

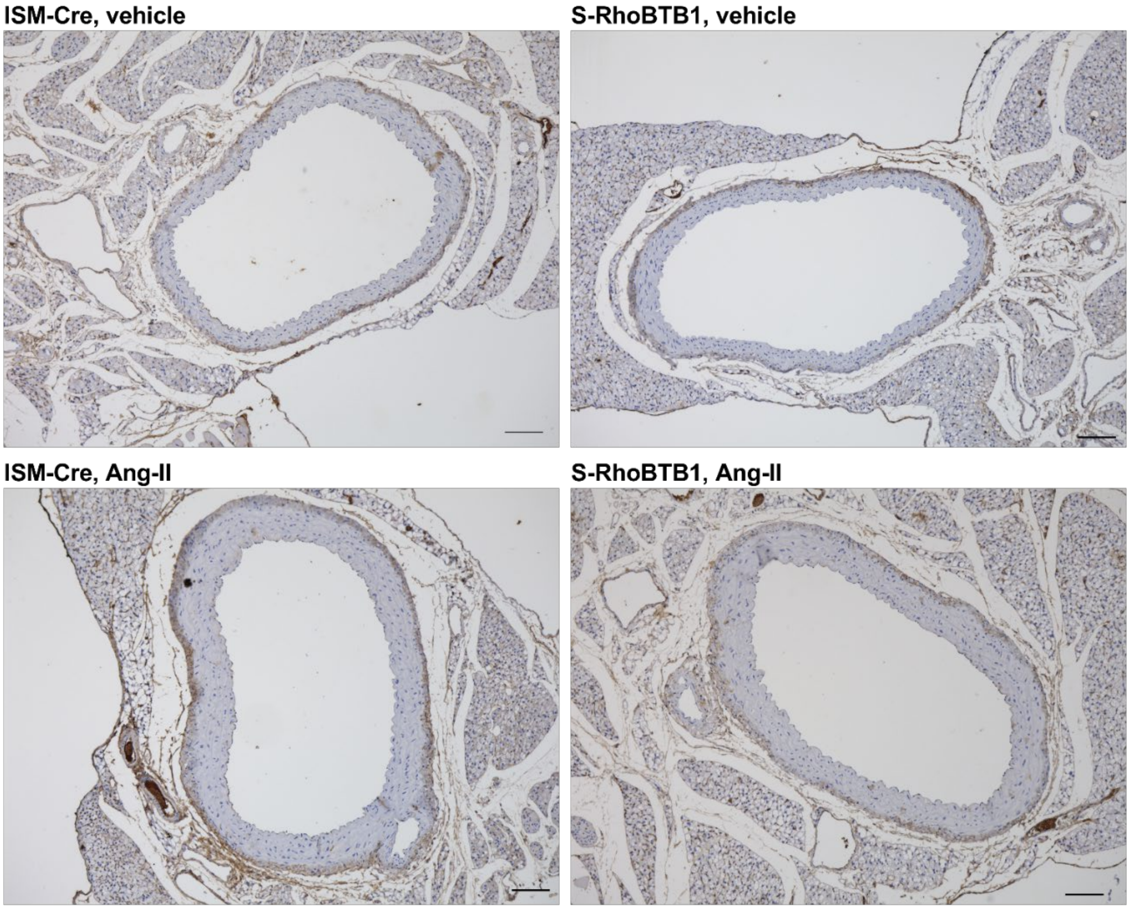

Supplemental Figure 5. Advanced Glycation End Product (AGEs) Staining.

Representative immunohistochemistry staining of AGEs in the aortae cross-sections from indicated animals 2-weeks after the completion of Tamoxifen injections (n= 6-9). Scale bar = 100 μm.

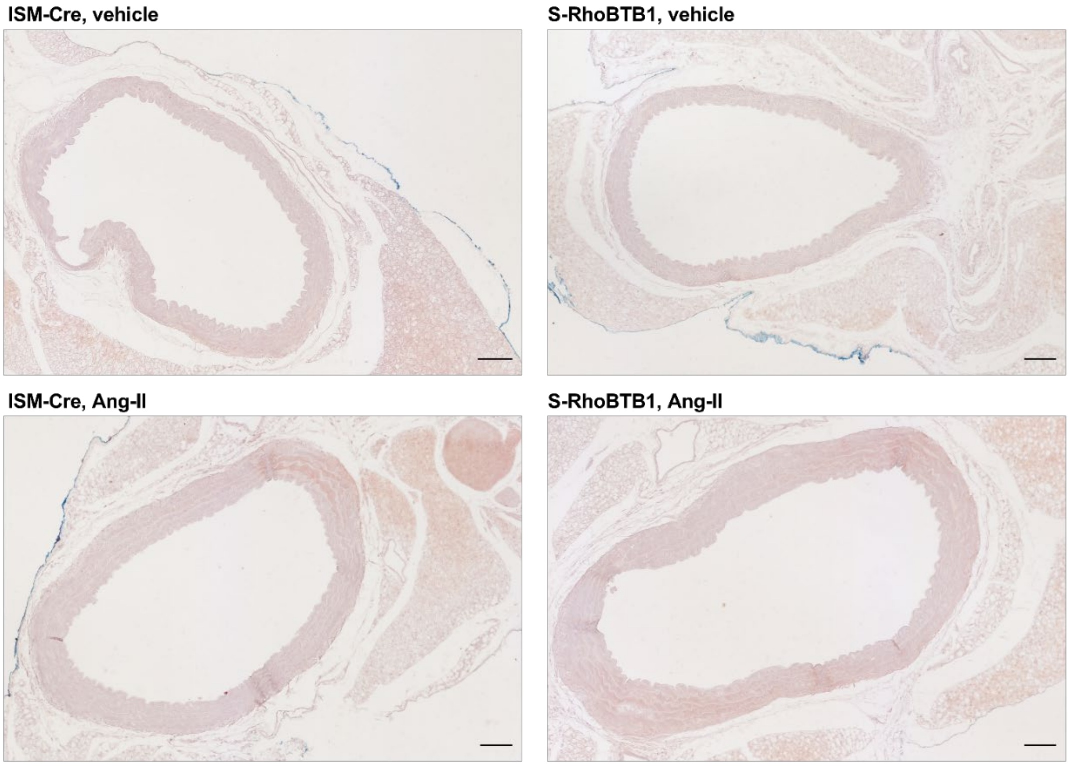

Supplemental Figure 6. Calcification.

Calcification was assessed by the Alizarin staining in the aortic cross-sections in the indicated groups (n=6-9).

Scale bar = 100 μm.

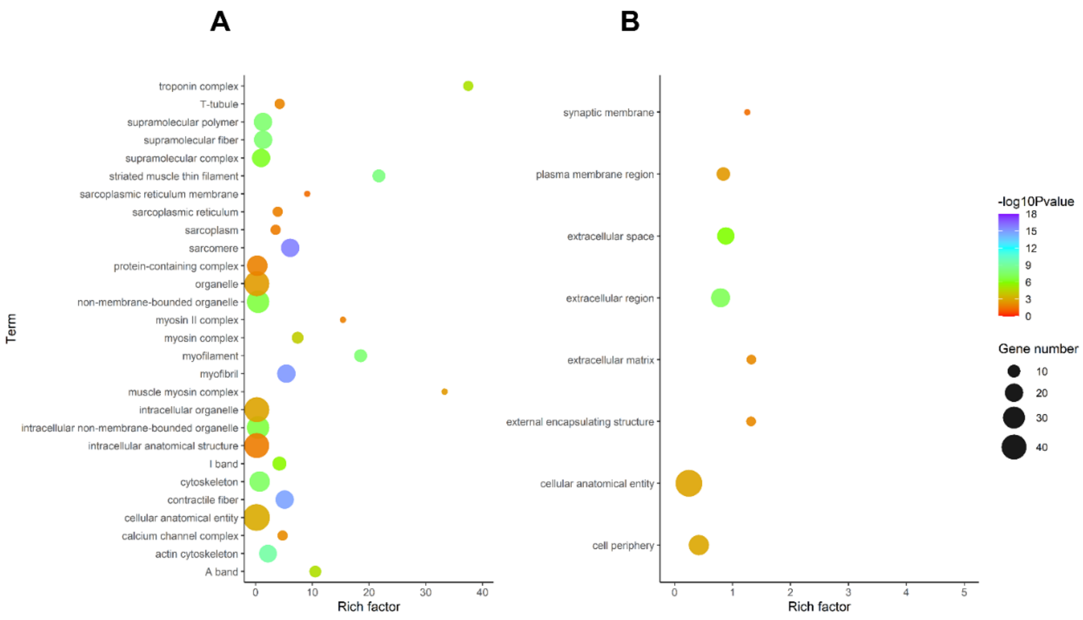

Supplemental Figure 7. Pathway Enrichment Analysis.

Pathway enrichment analysis of the genes that were either downregulated (A) or upregulated (B) by Ang-II and restored upon RhoBTB1. Statistical significance (p-value) of each ontology term was shown in different colors (see color key in the scale of -log10). Gene number reflects the number of DE genes from the meta-analysis intersected with the term and was depicted by different sizes of circles. Rich factor was calculated by dividing the number of DE genes by the total number of genes in each term.

Full unedited gel for Figure 1C: tdTomato western blot

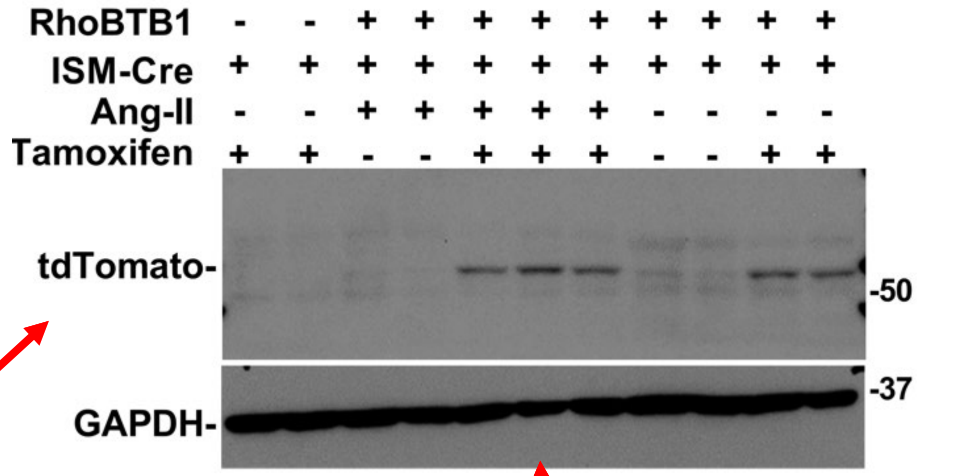

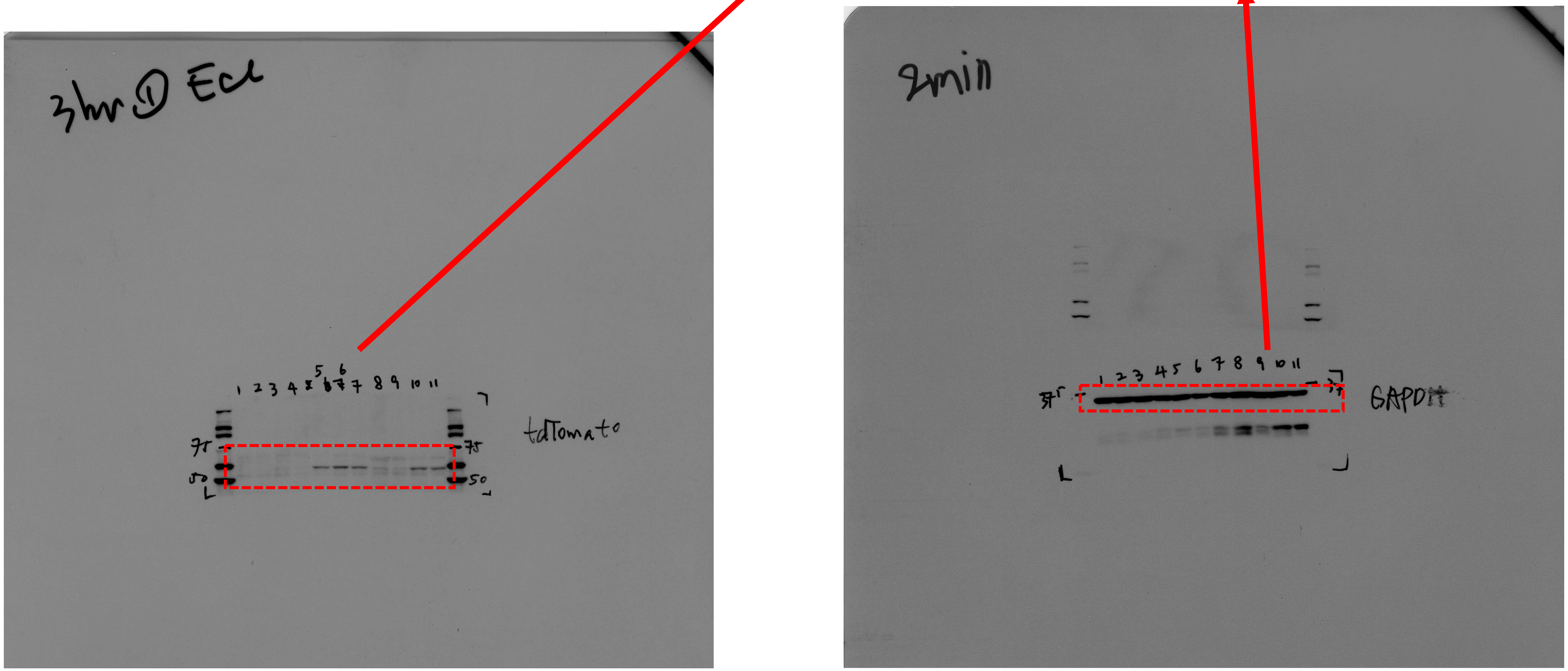

Full unedited gel for Figure 8A: Actin polymerization western blot

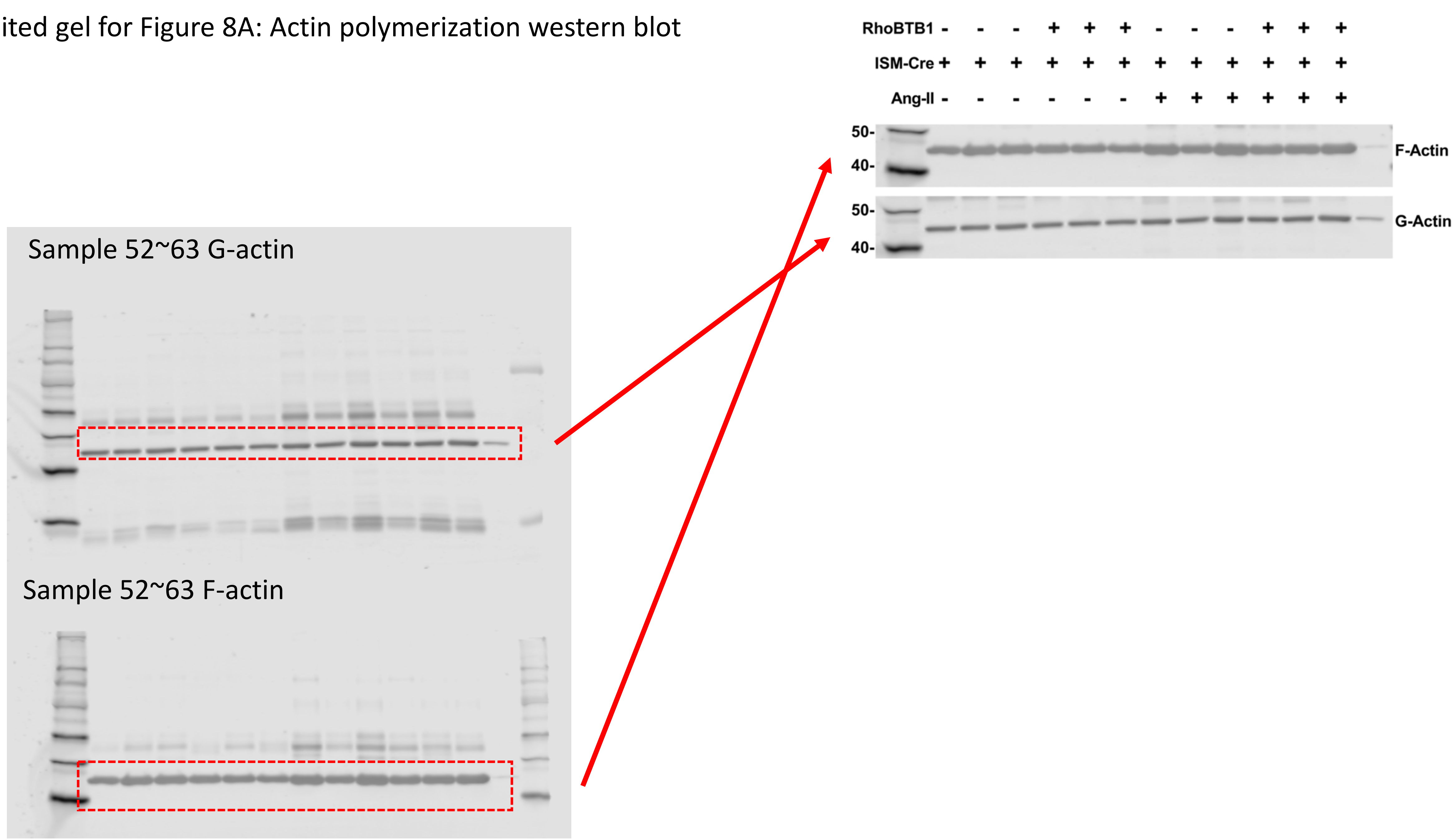

Data for Figure 8B

These are independent biological replicates for the WB in Figure 8A which are not shown, but are part of the quantification in 8B.

Sample 39~50 G-actin

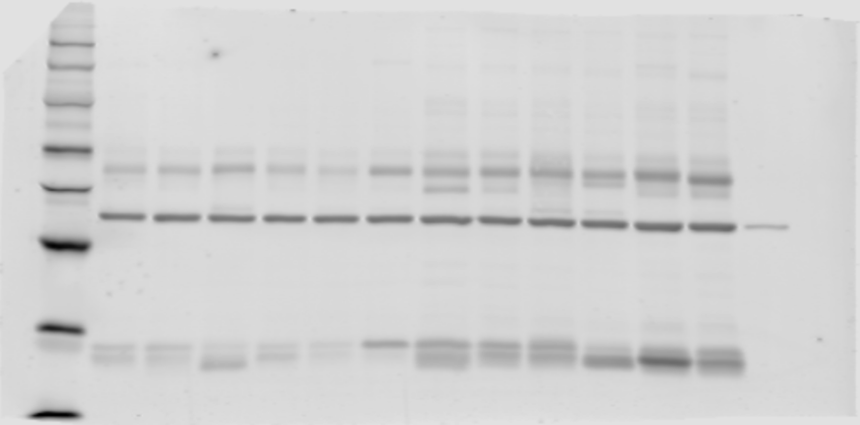

Sample 39~50 F-actin

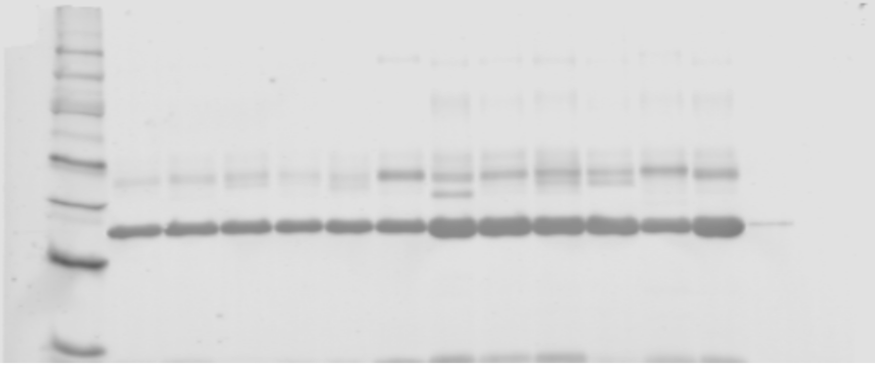

Sample 64~72 G-actin

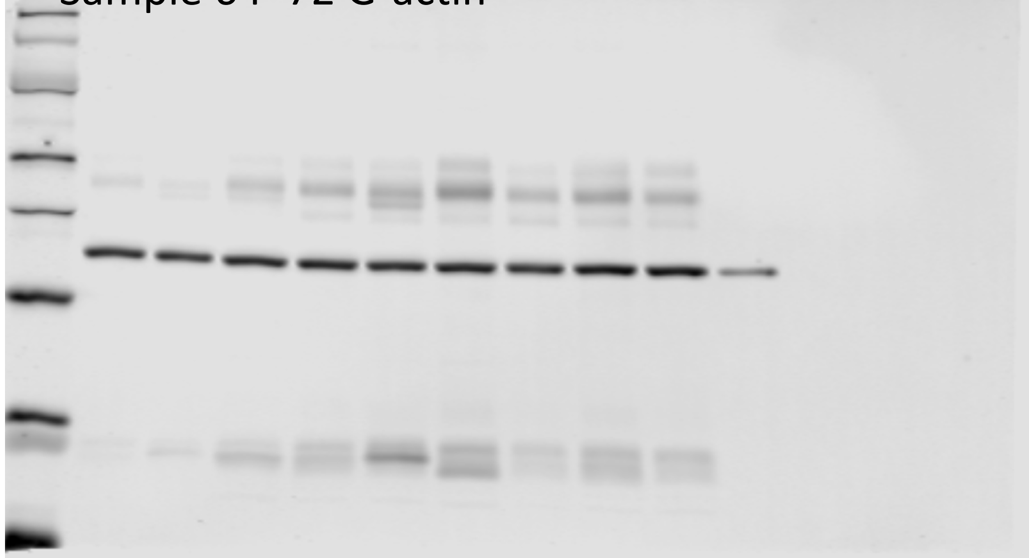

Sample 64~72 F-actin

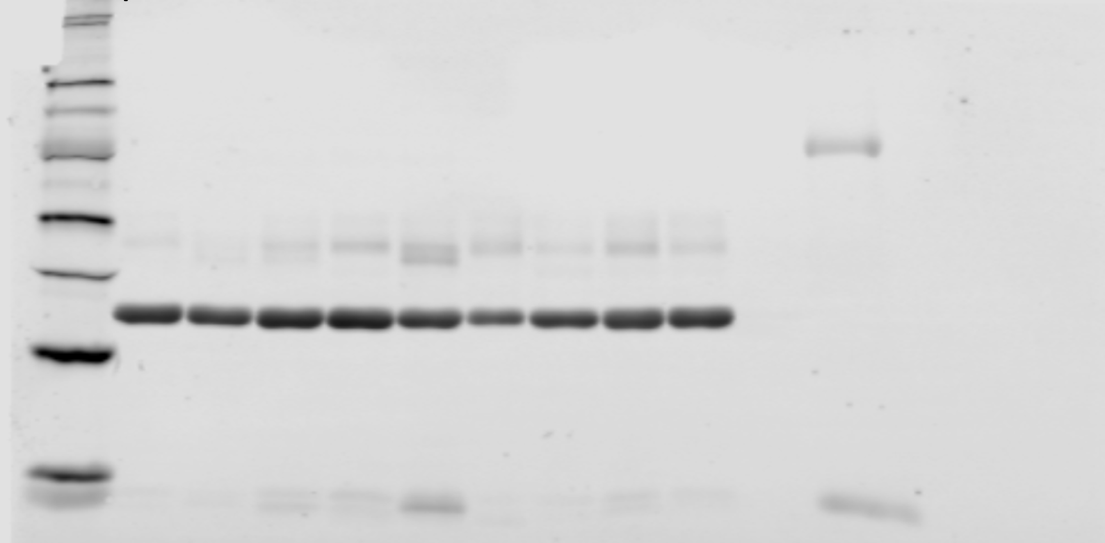

Full unedited gel for Figure 9A: phosphorylated/total Cofilin western blot

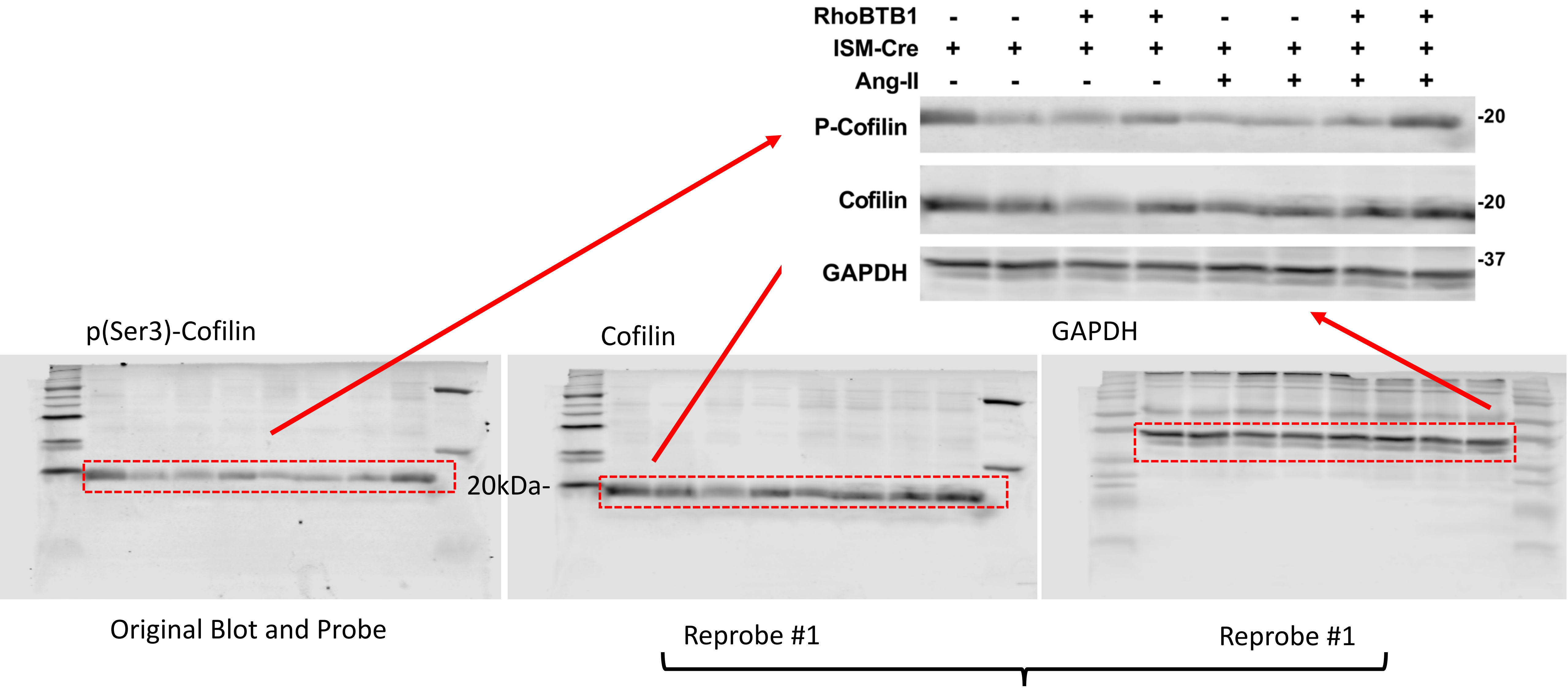

Probes simultaneous with rabbit (cofilin) and mouse (GAPDH) primaries and different species secondary with different fluors.

Data for Figure 9A: Independent biological replicates for the WB in Figure 9A (not shown), part of the quantification in 9B.

p(Ser3)-Cofilin | Cofilin | GAPDH

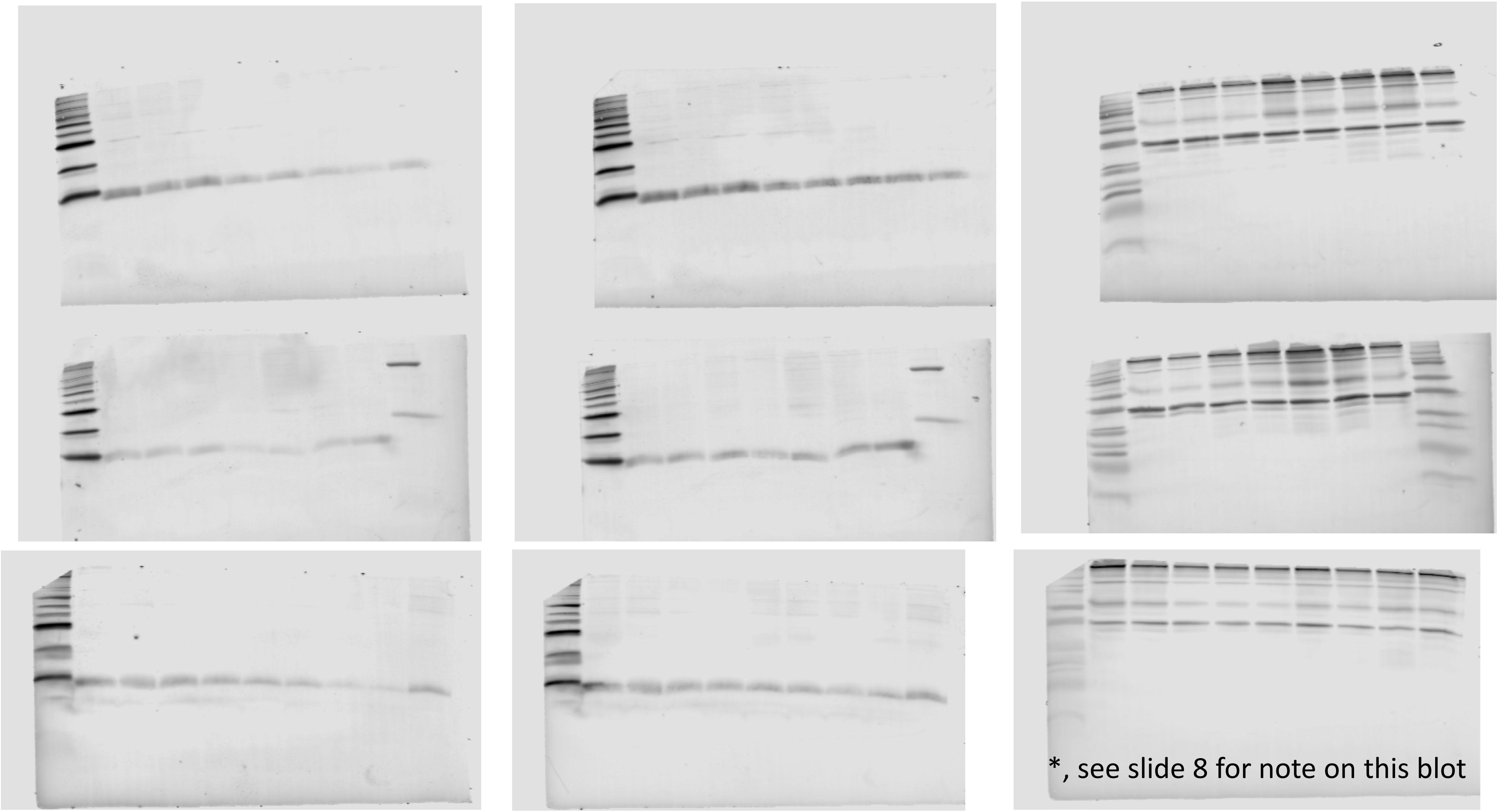

Full unedited gel for Figure 9B (upper left) and biological replicates for quantification (gels 2-4)

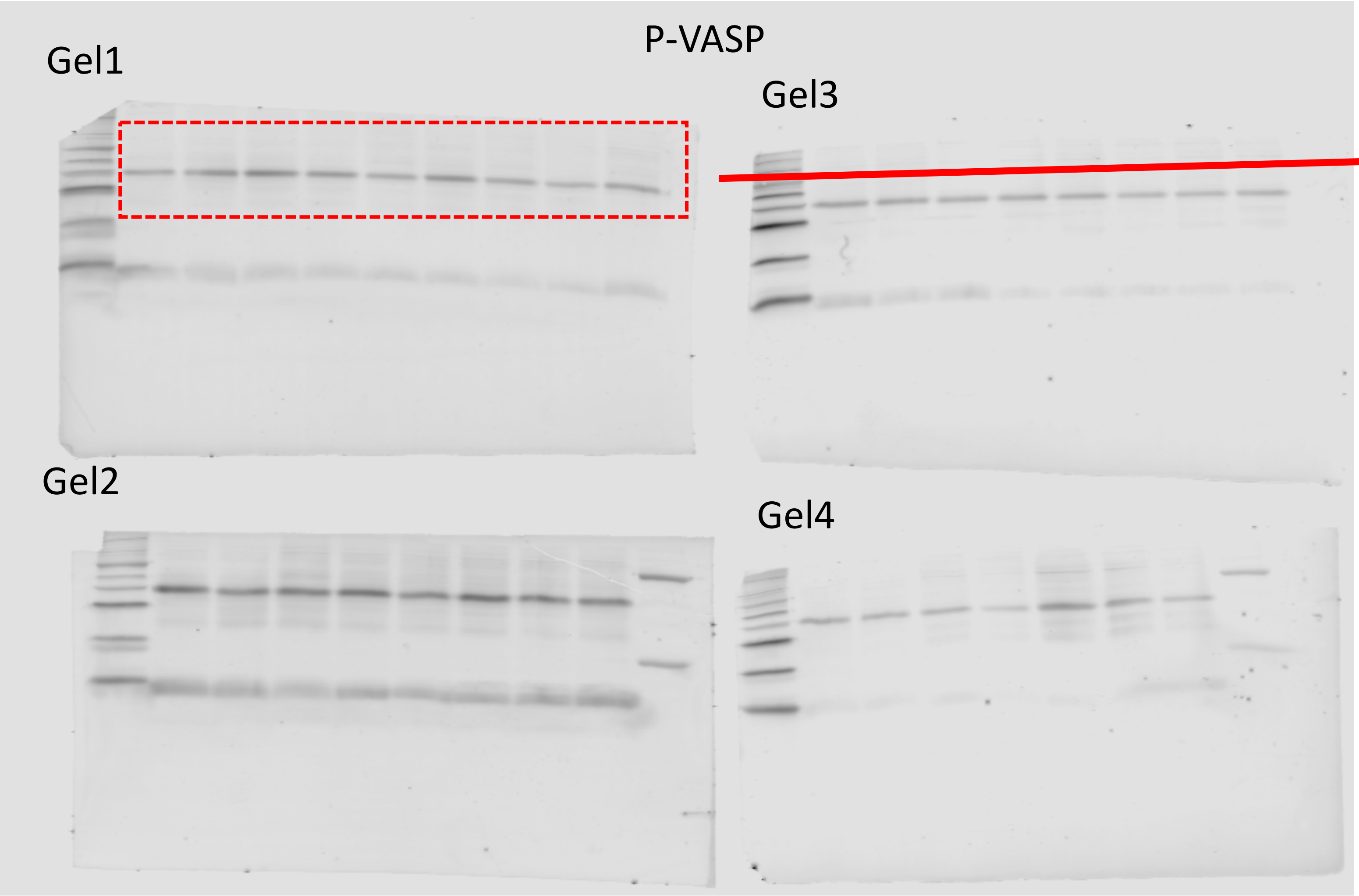

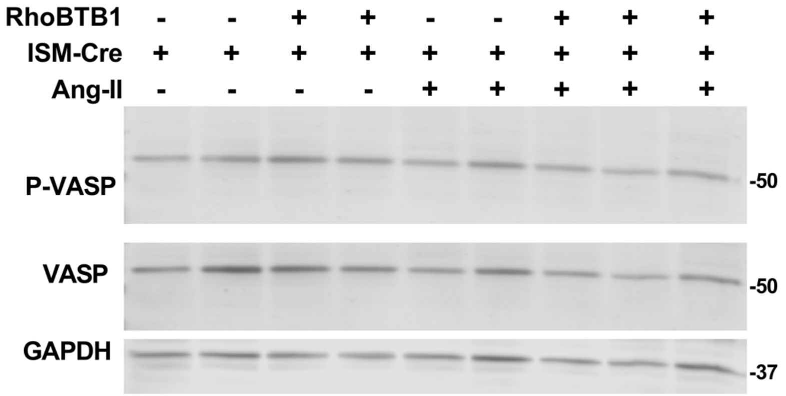

Original antisera for initial probe was P-VASP

Full unedited gel for Figure 9B (upper left) and biological replicates for quantification (gels 2-4)

Total VASP

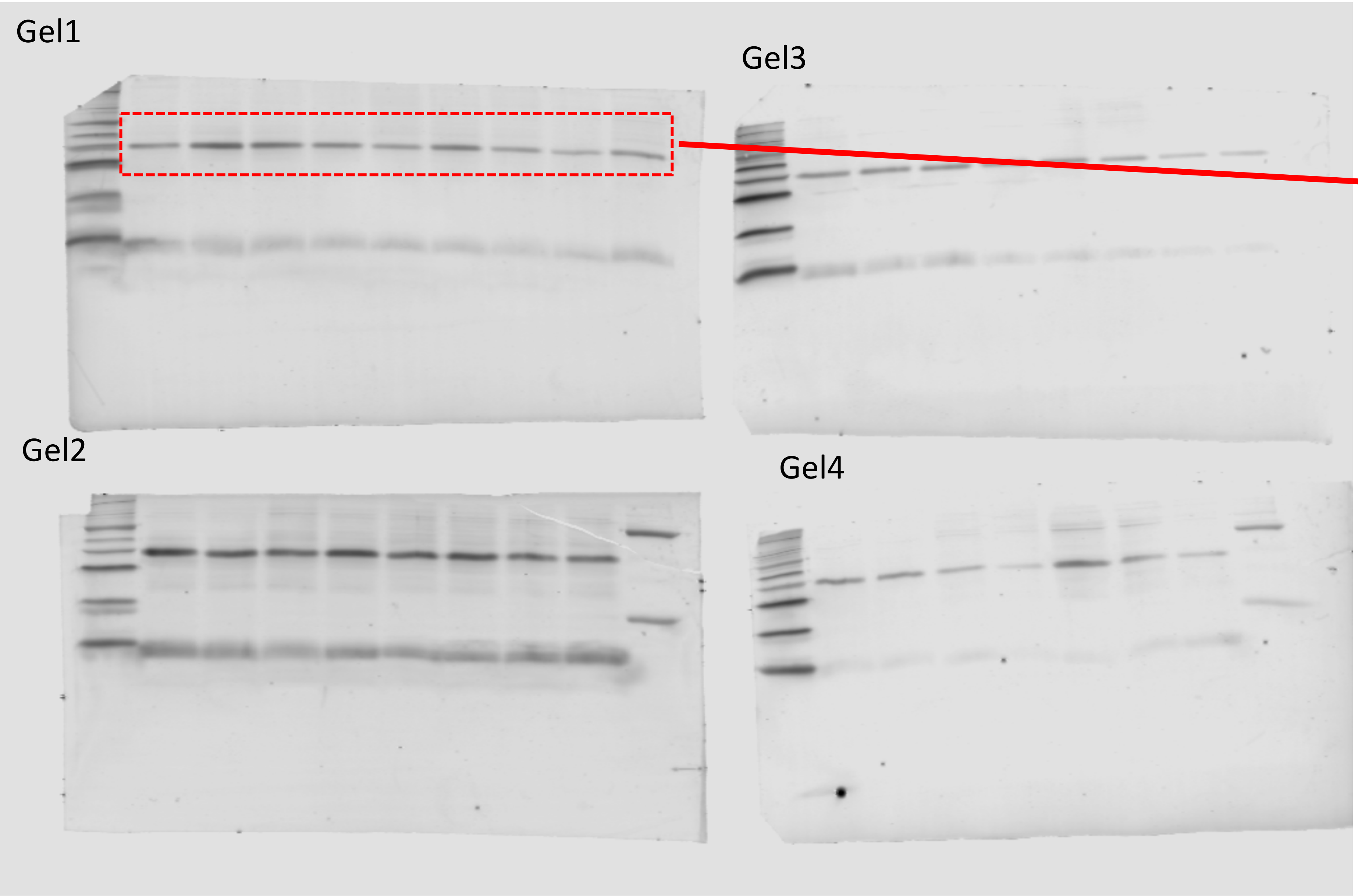

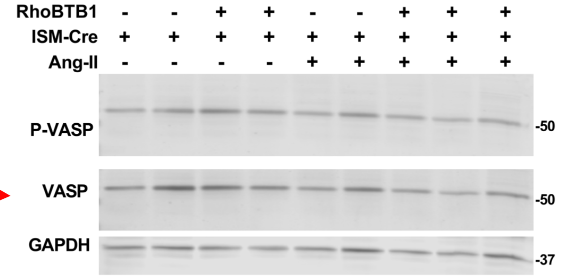

Reprobe of blots from previous page for Total VASP

Full unedited gel for Figure 9B (upper left) and biological replicates for quantification (gels 2-4)

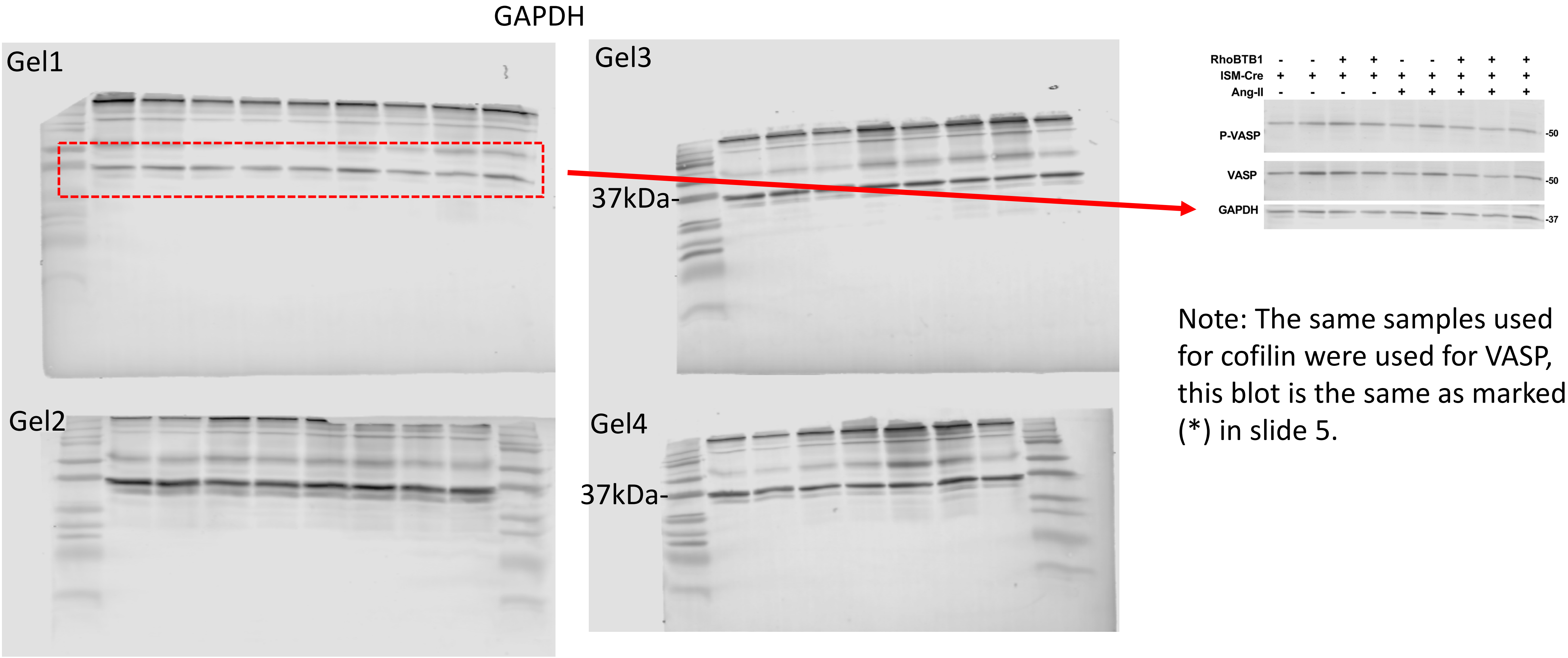

Note: The same samples used for cofilin were used for VASP, this blot is the same as marked (*) in slide 5.
